# Domain binding and isotype dictate the activity of anti-human OX40 antibodies

**DOI:** 10.1136/jitc-2020-001557

**Published:** 2020-12-21

**Authors:** Jordana Griffiths, Khiyam Hussain, Hannah L Smith, Theodore Sanders, Kerry L Cox, Monika Semmrich, Linda Mårtensson, Jinny Kim, Tatyana Inzhelevskaya, Chris A Penfold, Alison L Tutt, C Ian Mockridge, HT Claude Chan, Vikki English, Ruth F French, Ingrid Teige, Aymen Al-Shamkhani, Martin J Glennie, Bjorn L Frendeus, Jane E Willoughby, Mark S Cragg

**Affiliations:** 1 Antibody and Vaccine Group, Centre for Cancer Immunology, Cancer Sciences Unit, Faculty of Medicine, University of Southampton, Southampton, UK; 2 Preclinical Research, BioInvent International AB, Lund, Sweden

**Keywords:** costimulatory and Inhibitory t-cell receptors, immunotherapy, t-lymphocytes, immunomodulation

## Abstract

**Background:**

Previous data suggests that anti-OX40 mAb can elicit anti-tumor effects in mice through deletion of Tregs. However, OX40 also has powerful costimulatory effects on T cells which could evoke therapeutic responses. Human trials with anti-OX40 antibodies have shown that these entities are well tolerated but to date have delivered disappointing clinical responses, indicating that the rules for the optimal use of anti-human OX40 (hOX40) antibodies is not yet fully understood. Changes to timing and dosages may lead to improved outcomes; however, here we focus on addressing the role of agonism versus depleting activity in determining therapeutic outcomes. We investigated a novel panel of anti-hOX40 mAb to understand how these reagents and mechanisms may be optimized for therapeutic benefit.

**Methods:**

This study examines the binding activity and in vitro activity of a panel of anti-hOX40 antibodies. They were further evaluated in several in vivo models to address how isotype and epitope determine mechanism of action and efficacy of anti-hOX40 mAb.

**Results:**

Binding analysis revealed the antibodies to be high affinity, with epitopes spanning all four cysteine-rich domains of the OX40 extracellular domain. In vivo analysis showed that their activities relate directly to two key properties: (1) isotype—with mIgG1 mAb evoking receptor agonism and CD8+ T-cell expansion and mIgG2a mAb evoking deletion of Treg and (2) epitope—with membrane-proximal mAb delivering more powerful agonism. Intriguingly, both isotypes acted therapeutically in tumor models by engaging these different mechanisms.

**Conclusion:**

These findings highlight the significant impact of isotype and epitope on the modulation of anti-hOX40 mAb therapy, and indicate that CD8+ T-cell expansion or Treg depletion might be preferred according to the composition of different tumors. As many of the current clinical trials using OX40 antibodies are now using combination therapies, this understanding of how to manipulate therapeutic activity will be vital in directing new combinations that are more likely to improve efficacy and clinical outcomes.

## Introduction

The use of immunomodulating monoclonal antibodies (mAb) to generate anti-tumor immune responses offers an exciting approach to cancer immunotherapy. mAb against immune checkpoint inhibitors such as ipilimumab and nivolumab, which target the co-inhibitory receptors CTLA-4 and PD-1, respectively, pioneered this approach and have demonstrated success in treating a number of previously untreatable cancers.^1 2^ However, many patients do not respond to these reagents and additional therapeutic strategies are required. Agonistic mAb targeting co-stimulatory receptors have emerged as targets for clinical development, in particular, tumor necrosis factor receptors (TNFR) superfamily members such as CD40,^3^ 4-1BB,^4^ and OX40.^5–7^ However, Freeman *et al*^8^ identified an intratumoral Treg signature which included TNFR family members with the hypothesis that they could be targeted instead by depleting antibodies in order to generate therapy. TNFR family members are typically characterized by an extracellular domain (ECD) consisting of several cysteine-rich domains (CRDs) which allow for binding of their respective trimeric ligands leading to receptor clustering and downstream signaling.^9^ mAb targeting such receptors have been shown to depend on their interaction with the inhibitory FcγR (FcγRIIB) to generate sufficient cross-linking and resultant agonistic activity.^10 11^ More recently, the ability of a number of TNFR mAb to cause deletion of Tregs via engagement of activatory FcγR has been demonstrated.^12 13^ The anti-mouse OX40 mAb, OX86, has previously been shown to enhance effector T-cell proliferation and survival leading to successful therapeutic outcomes in pre-clinical models.^5 14^ Recently, it has also been demonstrated to be capable of deleting Tregs in an activatory FcγR-dependent manner.^12^ This effect was directly influenced by isotype, with mIgG2a showing greater depleting capacity than the native rIgG1 isotype. Interestingly, Tregs were preferentially deleted over effector T cells which correlated with mOX40 expression on these cells.^12^

Work on several TNFRs has further highlighted the importance of the region targeted by the antibody in influencing the type and strength of effector function.^15–17^ For anti-CD40 mAb, the membrane distal CRD1-binding mAb were shown to be strong agonists of CD40 with membrane proximal mAb less potent.^16^ Furthermore, mAb binding CRD2-4 blocked CD40L and were potent antagonists. Additionally, anti-4-1BB mAb, which bound membrane proximal domains, engaged in more effective complement-dependent cytotoxicity and antibody-dependent cellular cytotoxicity killing mechanisms with antibody-dependent cellular phagocytosis less affected.^15^ Moreover, Zhang *et al*^17^ reported that mAb binding to mouse (m)OX40, which blocked ligand binding and bound CRD2, or bound at the membrane proximal domain (CRD4), provide stronger agonistic and anti-tumor activity than mAb binding CRD1 and 3. These results differed from those seen for hCD40, highlighting that the functional effects of mAb domain binding are likely to require assessment for each of the TNFR family members and validation for each species.

Given these discrepancies, we explored the optimal domain binding and isotype for a novel panel of anti-human (h)OX40 mAb which collectively bound to all four CRDs of the ECD of hOX40. We evaluated their function in vitro and in vivo as both mIgG1 and mIgG2a isotypes. Using a novel hOX40 knock-in (KI) mouse, we found that mIgG1 mAb were agonistic and engendered memory responses, whereas mIgG2a mAb had depleting activity with poorer memory recall responses. The strength of these effector functions appeared to correlate with domain binding; those mAb which bound to the most membrane proximal domain (CRD4) which did not block ligand binding, showed the strongest agonistic activity as mIgG1 as well as the most potent depleting activity as mIgG2a. This data highlights how mAb to different TNFR (and different species) exhibit different requirements in relation to optimal domain binding and effector function, and indicate how more active anti-hOX40 mAb might be developed.

## Results

### hOX40KI mice express hOX40 and develop normally

**Figure 1:**
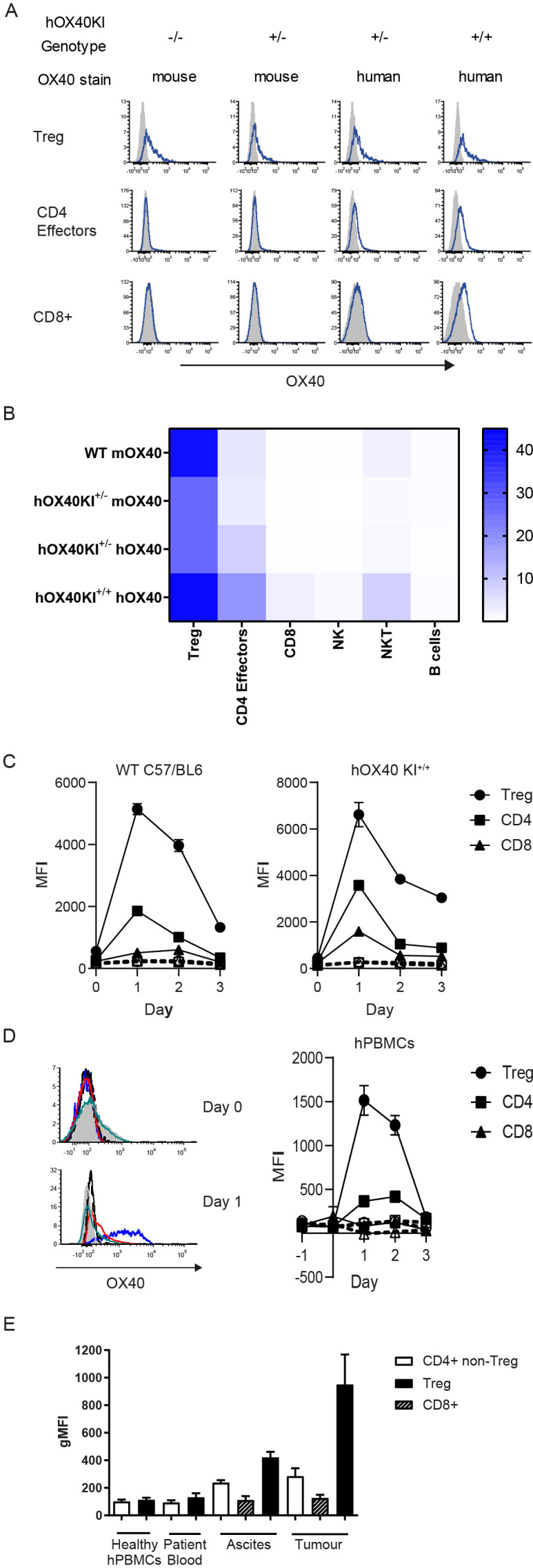
hOX40KI mice express hOX40 in a hierachial manner. (A). Expression of OX40 (mouse (m) and human (h)—blue line) compared with isotype control (shaded histogram) on Treg (top row), CD4+ effectors (middle row) and CD8+ T cells (bottom row). Representative plots shown. (B) Heat map summarizing OX40 expression as a percentage on resting mouse splenocytes (n=4). (C) Expression of mOX40 (left panel) and hOX40 (right panel) on splenocytes from WT or hOX40KI mice activated with αCD3 and αCD28 (n=3). Isotype controls showed as dashed lines. (D) Expression of hOX40 on hPBMCs activated with αCD3 and αCD28. Histograms show hOX40 expression on Tregs (blue line, isotype control black line), CD4+ (red line, isotype control black dashed line) and CD8+ (green line, isotype control gray filled histogram) from a representative donor on Day 0 (top panel) and Day 1 (bottom panel). Line graph (right panel) shows average expression, isotype controls shown as dashed lines (n=3). (E) hOX40 expression on CD4+ effector T cells (white bars), CD8+ T cells (hatched bars) or Tregs (black bars) isolated from healthy donors or blood, ascites and tumor from cancer patients (n=4–16). MFI, mean fluorescence intensity; gMFI, geoMean fluorescence intensity; hPBMC, human peripheral blood mononuclear cells; KI, knock-in; WT, wildtype.

To investigate the immunotherapeutic potential of a new panel of anti-hOX40 mAb, we generated a new KI mouse, designed to express hOX40 ECD and mOX40 transmembrane and intracellular domains (online supplemental figure 1A). PCR confirmed integration of the construct and identified wildtype (WT), hOX40KI^+/−^ and hOX40KI^+/+^ mice (online supplemental figure 1B). Analysis of OX40 surface expression (mouse and human) on resting splenocyte T cells from WT, hOX40KI^+/−^ and hOX40KI^+/+^ mice confirmed that the chimeric receptor was expressed at the cell surface on relevant cell types (figure 1A and B) and in a gene dose-dependent manner (online supplemental figure 1C). Expression of OX40 in all three genotypes was largely restricted to T-cell lineages (figure 1A and B and online supplemental figure 1D). In line with previous reports,^12 18–20^ a hierarchal expression pattern among the T-cell subsets was observed in all genotypes, with expression highest on Tregs followed by CD4+ effectors, with limited expression on resting CD8+ T cells (figure 1A and B). To address whether the hOX40KI^+/+^ mice represented a functional model to study the OX40L:OX40 axis, we performed surface plasmon resonance (SPR) analysis of mOX40L and hOX40L binding to hOX40 (online supplemental figure 1E). Both mOX40L and hOX40L bound similarly to hOX40, in agreement with earlier studies, showing that mOX40L engages the same domains on hOX40 as hOX40L.^21^ Furthermore, OX40 knock-out (KO) mice are reported to have a subtle defect in Treg numbers (reviewed in^22^). However expression of the chimeric receptor did not affect normal immune cell development (online supplemental figure 1F), further indicating that OX40L:OX40 signaling axis is intact in these hOX40KI mice.

10.1136/jitc-2020-001557.supp1 Supplementary data

Consistent with previous findings,^23^ activated splenocytes isolated from WT, hOX40KI^+/−^ and hOX40KI^+/+^ mice showed peak OX40 expression between 24 and 48 hours post activation (figure 1C). The kinetics of expression of hOX40 on hOX40KI^+/−^ and hOX40KI^+/+^ splenocytes correlated with that of hOX40 on activated human peripheral blood mononuclear cells (hPBMCs) (figure 1D). A hierarchy of expression was also observed in both activated splenocytes and hPBMCs with greatest expression detectable on Tregs (figure 1C and D). Samples taken from human cancer patients also showed the same pattern (Tregs >CD4+effectors > CD8+T cells) with highest OX40 expression on T cells isolated from tumor sites (figure 1E). Collectively, these results validated the use of the hOX40KI model to assess anti-hOX40 mAb.

### Generation and characterization of a panel of anti-hOX40 mAb

**Figure 2:**
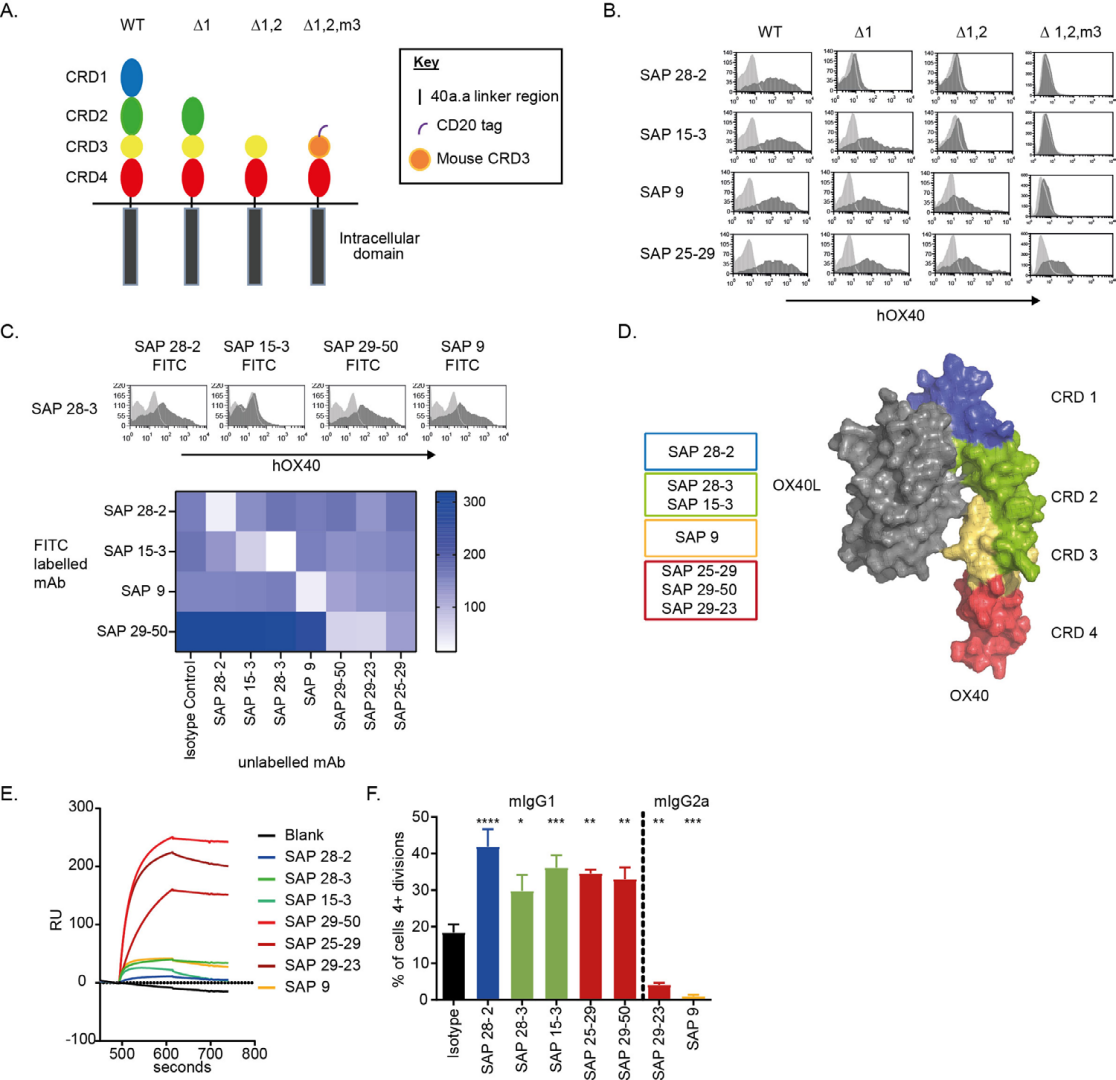
Characterization of a panel of anti-hOX40 mAb. (A) Schematic of the WT and domain mutant hOX40 constructs generated. CRD3 from mOX40 was used to stabilize the human CRD4 construct. (B) Binding of anti-hOX40 mAb to domain constructs detected by a PE-labeled secondary Fab_2_ fragments. Representative histograms show hOX40 mAb binding (dark gray histogram) compared with an isotype control (light gray histogram). (C) Representative histograms show anti-hOX40 FITC-labeled mAb binding (dark gray histogram) in relation to an isotype control (light gray histogram) after binding of unlabeled anti-hOX40 mAb. The heat map shows MFI of FITC-labeled antibody binding in the presence of unlabeled antibodies with the absence of color indicating blocking. (D) Diagram summarizing antibody binding domains in relation to the crystal structure of the OX40:OX40L complex. (E) Surface plasmon resonance analysis of anti-hOX40 mAb binding to hOX40-hFc in the presence of hOX40L-His fusion protein. (F) Proliferation of hCD8+ T cells within PBMC cultures in response to sub-optimal anti-CD3 and anti-hOX40 mAb stimulation (representative of four individual donors). Mean±SEM ****p<0.0001, ***p<0.001, **p<0.01, *p<0.05, Dunnett’s multiple comparison test. PE, Phycoerythrin; MFI, mean fluorescence intensity; CRD, cysteine-rich domain; FITC, fluorescein isothiocyanate; mAb, monoclonal antibody; PBMC, peripheral blood mononuclear cells; WT, wildtype.

A panel of seven anti-hOX40 mAb were subsequently generated by conventional hybridoma technology and characterized. All mAb displayed a high affinity for hOX40 (KD values between 10^−9^ and 10^−10^M) as determined by SPR (online supplemental figure 2A) and did not bind mOX40 (online supplemental figure 2 B and C). The crystal structure of hOX40 indicates four CRDs.^21^ To determine which of these the anti-hOX40 mAb bound, hOX40 domain mutants were generated, lacking CRD1, CRD1+2 or CRD1, 2+3 (figure 2A). A hCD20 epitope tag and mOX40 CRD3 domain were added to the final construct to stabilize its expression. Across the panel, at least one antibody bound to each of the different domains (figure 2B and online supplemental figure 2D). Cross-blocking experiments showed that anti-hOX40 mAb which bound to the same domain blocked the binding of one another, whereas mAb binding to different CRDs could bind simultaneously (figure 2C). The domain binding for each antibody is summarized in figure 2D. Finally, SPR analysis revealed that only mAb binding to CRD4 were able to bind in the presence of the ligand (figure 2E and online supplemental figure 2E), indicating ligand binding blocks binding of mAb that recognize epitopes in CRD1−3.

10.1136/jitc-2020-001557.supp4 Supplementary data

Antibodies were then assessed for their ability to augment suboptimal anti-CD3-mediated proliferation of hPBMC. All of the mIgG1 mAb increased proliferation of CD8+ T cells (figure 2F) whereas mIgG2a mAb reduced proliferation. Differences between mIgG1 and mIgG2a isotypes have previously been reported for other TNFR family members^24 25^ and so we class-switched the mAb so that both mIgG1 and mIgG2a isotypes were available. A similar trend was seen in hOX40KI splenocytes stimulated with anti-hOX40 mAbs with mIgG1 mAb showing a trend towards an increase in proliferation while mIgG2a mAb showed a clear reduction in proliferation (online supplemental figure 3).

10.1136/jitc-2020-001557.supp5 Supplementary data

### Anti-hOX40 mIgG1 mAb are agonistic in vivo

**Figure 3:**
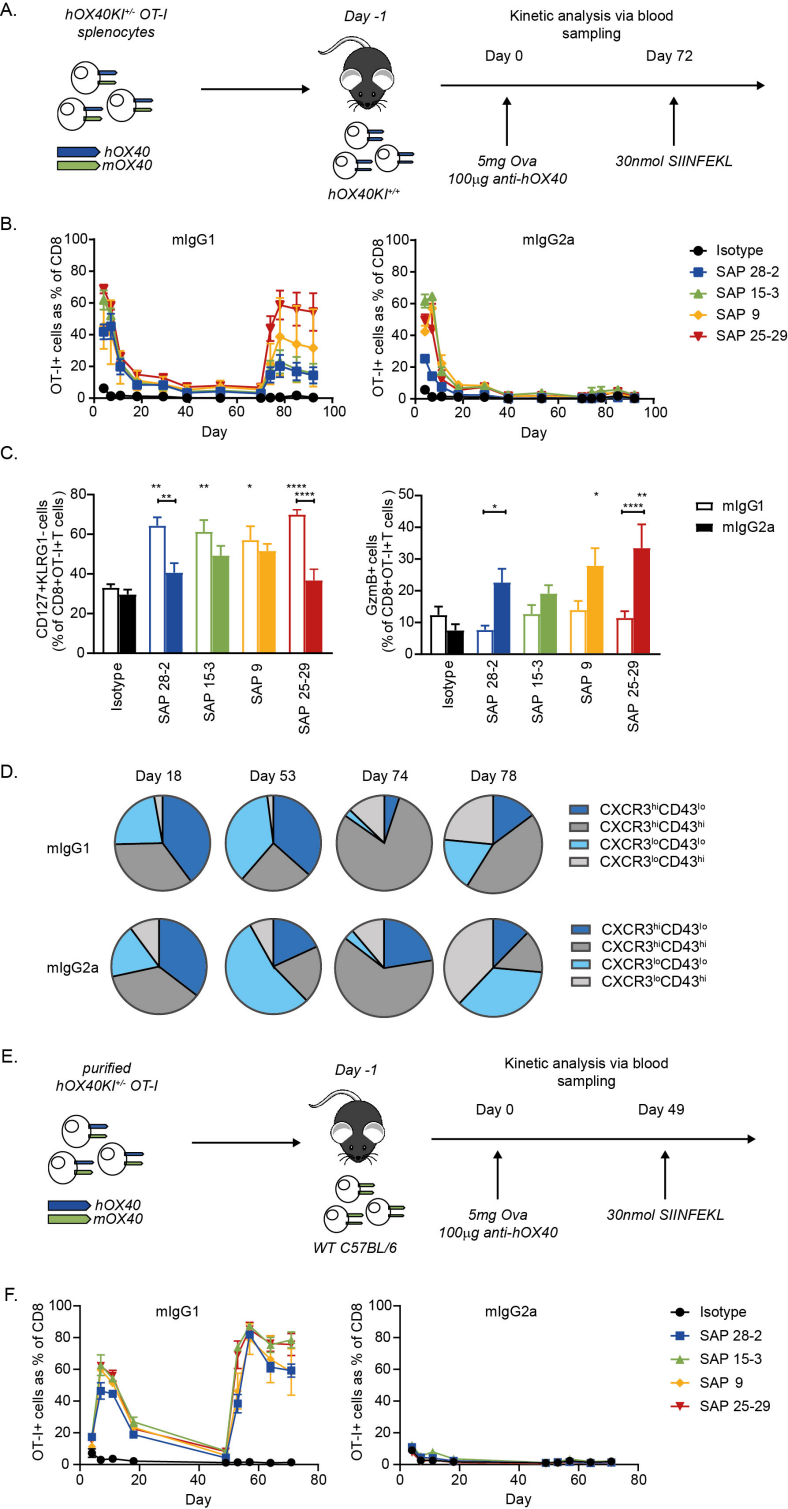
Anti-hOX40 mIgG1 act agonistically in vivo. (A) Schematic of the OT-I model used in B−D. 1×10^5^ hOX40KI^+/−^ OT-I cells were transferred into hOX40KI^+/+^ recipients. Mice were challenged with 5 mg OVA and 100 µg of antibody. (B). Kinetic analysis of OT-I expansion in response to anti-hOX40 mIgG1 mAb (left panel) or mIgG2a mAb (right panel) (n=4 representative of two independent experiments). (C) Analysis of memory and effector phenotyping of OT-I T cells in blood at Day 18. MPECs—CD127+ KLRG1− (left panel) and granzyme B+ OT-I T cells (right panel) (n=4 isotype controls and n=8 all treatment groups, pooled from two independent experiments). (D) CXCR3/CD43 profiling of OT-I T cells in blood at time points indicated in response to SAP 25–29 stimulation (n=4). (E) Schematic of the OT-I model used in F. 1×10^5^ hOX40KI^+/−^ OT-I were transferred into WT C57BL/6 recipients. Mice were challenged with 5 mg OVA and 100 µg of antibody. (F) Kinetic analysis of OT-I expansion in response to anti-hOX40 mIgG1 mAb (left panel) or mIgG2a mAb (right panel) (n=4 representative of two independent experiments). Mean±SEM ****p<0.0001, **p<0.01 *p<0.05, Sidak’s multiple comparison test. mAb, monoclonal antibody; MPEC, memory precursor cells; WT, wildtype.

To investigate the ability of the anti-hOX40 mAb to cause antigen-specific CD8+ T-cell expansion in vivo, we used the OT-I model whereby antigen-specific T cells are transferred into naive recipients. hOX40KI^+/−^ OT-I T cells, which recognize the OVA_257–264_/H-2K^b^ complex, were adoptively transferred into hOX40KI^+/+^ mice before vaccination with OVA and administration of anti-hOX40 mAb as either mIgG1 or mIgG2a (figure 3A). Both mIgG1 and mIgG2a anti-hOX40 mAb expanded antigen-specific CD8+ OT-I T cells in blood compared with OVA alone and to a similar extent with the exception of SAP 28–2 which was notably weaker as a mIgG2a (figure 3B and online supplemental figure 4A). Despite reaching similar frequencies at the peak of the primary response, on re-challenge with SIINFEKL peptide alone, a significantly smaller recall response was seen in mice that had received mIgG2a antibodies (figure 3B and online supplemental figure 4B). Given the time between antibody administration and re-challenge with SIINFEKL peptide, it is likely this lack of recall reflects responses initiated during the priming stage as opposed to any effects of mAb persisting from the initial challenge. The frequency of OT-I cells pre-recall in mIgG1-treated mice strongly correlated with the strength of the recall peak (online supplemental figure 4C); however due to the lack of a recall response seen in the mIgG2a it is not possible to determine if this is also the case for the mIgG2a isotype. These data suggest that the number of OT-I cells present before re-challenge is a key determining factor for the strength of the recall response and that mIgG1 and mIgG2a mAb deliver signals during the primary response which results in different resting memory populations.

10.1136/jitc-2020-001557.supp6 Supplementary data

To understand this difference in memory response, OT-I T cells in the blood were phenotyped during the primary and memory stages. Analysis of CD127 and KLRG1 expression during the primary response can identify short-lived effector cells (SLECs—CD127− KLRG1+) and memory precursor cells (MPECs—CD127+ KLRG1−).^26^ The frequency of MPECs was higher in the mIgG1 groups at Day 18 (figure 3C). While frequencies of SLECs in the blood was similar between isotypes (online supplemental figure 4D) granzyme B production was higher in SAP 9 and 25–29 mIgG2a-treated mice compared with mIgG1-treated mice (figure 3C). Additionally, these data indicated that there may be a domain-related trend in granzyme B production in those mice receiving mIgG2a mAb, with the following hierarchy; CRD4 binding mAb (SAP 25–29)>CRD3 (SAP 9)>CRD2 (SAP 15–3) and CRD1 (SAP 28–2). In infection models, the relative frequencies of each subpopulation (SLECs versus MPECs) in the primary response does not always correlate with the accumulation of CD8+ cells during a recall response.^26–28^ We therefore expanded our analysis to CXCR3 and CD43, shown to define three distinct populations of memory cells with a hierarchy of recall response (CXCR3^hi^CD43^lo^ > CXCR3^hi^CD43^hi^ > CXCR3^lo^CD43^lo^).^29 30^ Mice that had been treated with mIgG1 anti-hOX40 mAb gave rise to a higher frequency of CXCR3^hi^CD43^lo^ and CXCR3^hi^CD43^hi^ cells in comparison to mice that had been treated with mIgG2a mAb (figure 3D and online supplemental figure 4E). This effect was most strongly seen with the SAP 25–29 CRD4-binding antibody (figure 3D and online supplemental figure 4E). This difference in frequencies between mIgG1-treated and mIgG2a-treated mice, while slight during the contraction phase (D18), became more evident during the resting memory period prior to re-challenge (D53). Immediately following re-challenge with SIINFEKL peptide (D74), both mIgG1 and mIgG2a groups displayed expansion of cells with high proliferative capacity, but further into the memory response (D78), the higher prevalence of cells with greater proliferative capacity in mIgG1-treated mice was re-established (figure 3D). Furthermore, mice treated with mIgG2a anti-hOX40 mAb had a higher frequency of effector-like memory cells (CXCR3^lo^CD43^lo^) in the resting memory phase (D53) (figure 3D). Again, this contrast between mIgG1-treated and mIgG2a-treated mice was re-established from D78 following re-challenge. These data indicate, that in our OT-I transfer model, the choice of isotype significantly impacts the development of a robust memory response.

These results also highlighted a discrepancy in the effect of the mIgG2a anti-hOX40 mAb between in vitro and in vivo experiments. mIgG2a anti-hOX40 mAb caused an inhibition of proliferation in vitro (figure 2F), whereas T-cell expansion was seen in vivo (figure 3B). Therefore, hOX40KI^+/−^ CD8+ OT-I T cells were purified and transferred into WT C57BL/6 mice to see if both mIgG1 and mIgG2a isotypes were capable of acting directly on CD8+ T cells (figure 3E). While mIgG1 mAb were able to drive a similar expansion as before, mIgG2a mAb had no effect (figure 3F). These results indicate that the mIgG2a mAb act indirectly, through non-CD8+ T cells, to facilitate OT-I expansion in contrast to the ability of mIgG1 to act directly on the OT-I T cells.

### Anti-hOX40 mIgG2a mAb deplete hOX40 expressing cells

**Figure 4:**
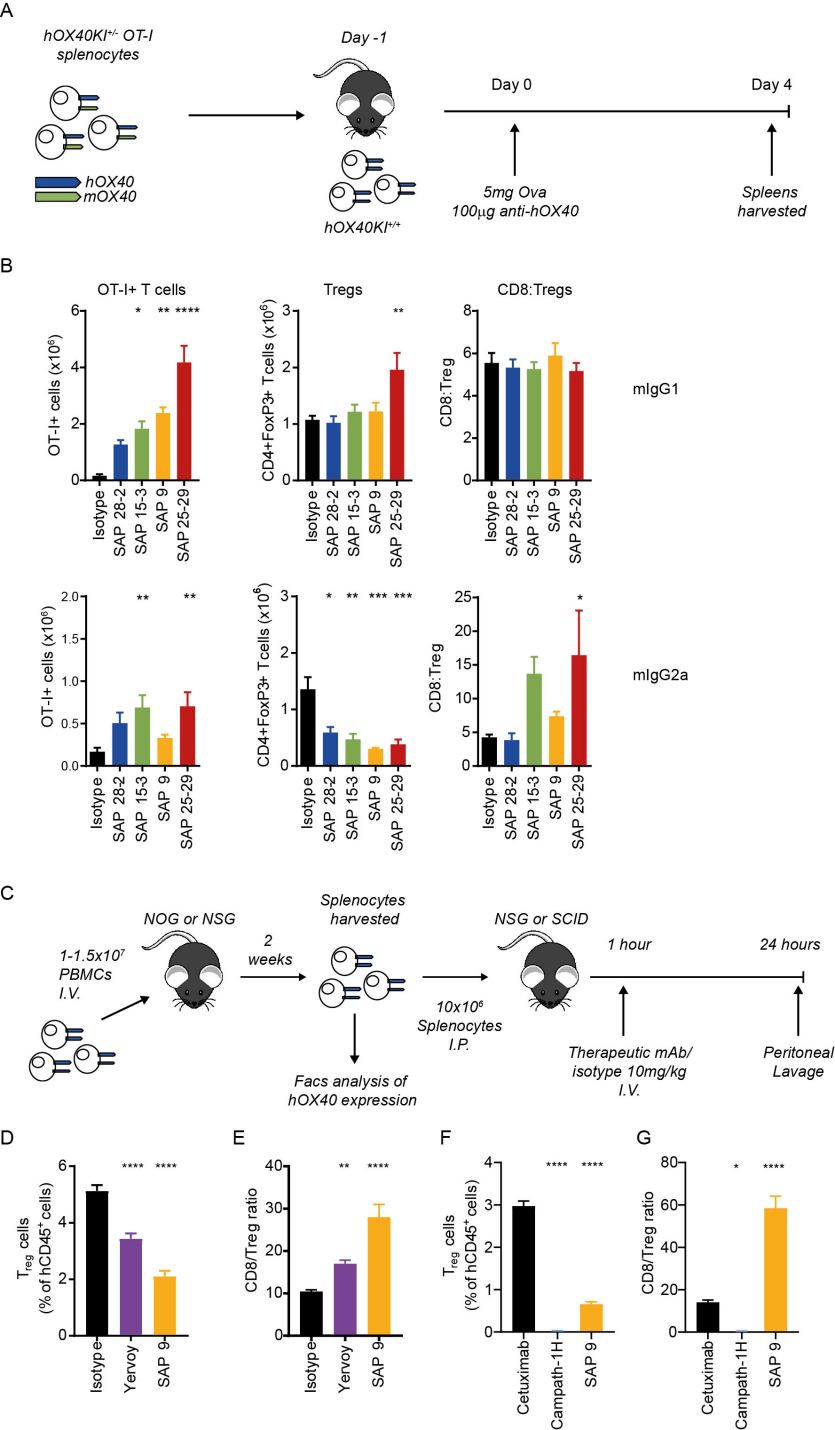
Anti-hOX40 mIgG2a mAb elicit Treg cell depletion in vivo. (A) Schematic of OT-I model used to assess cell depletion. (B) Assesment of Tetramer+ve (left panel), Treg cell numbers (middle panel) and CD8:Treg ratio (right panel) in response to anti-hOX40mAb either as a mIgG1 (top row) or mIgG2a (bottom row) (n=4 SAP 28–2, n=5 SAP 15–3 and SAP 9, n=6 all remaining groups, pooled from two independent experiments). (C) Schematic of the NSG/PBMC model to assess depletion of hPBMCs. 1.5×10^7^ (D, E) or 1×10^7^ (F, G) hPBMC were transferred into NOG (D, E) or NSG (F, G) mice. Two weeks later, splenocytes were harvested and transferred into SCID (D, E; n=11 SAP 9, n=12 Yervoy, n=13 isotype control pooled from two independent experiments) or NSG (F, G; n=7 pooled from two independent experiments) recipients which were then treated with depleting antibodies. Treg numbers (D, F) and CD8:Treg ratio (E, G) were determined by flow cytometry. Mean±SEM, ****p<0.0001, ***p<0.001, **p<0.01, *p<0.05. Sidak’s (B) and Tukey’s (F and G) multiple comparison test. FACS, fluorescence-activated cell sorting; hPBMC, human peripheral blood mononuclear cells; mAb, monoclonal antibody; NOG, NOD/Shi-*scid*/IL-2Rγ^null^; NSG, NOD scid gamma SCID, severe combined immune deficient.

We hypothesized that the indirect effect of mIgG2a mAb might involve deletion of Treg.^24^ Depletion of Tregs has previously been shown to allow greater expansion of OT-I and OVA responses.^31–33^ Therefore, to address this hypothesis, spleens were harvested from hOX40KI^+/+^ mice 4 days after treatment with either anti-hOX40 mIgG1 or mIgG2a mAb (figure 4A). Numeration of different T-cell subsets in the spleen showed that the mIgG2a mAb were able to significantly reduce the Treg population and to a lesser extent CD4+ effectors whereas mIgG1-treated mice evoked expansion of T-cell populations (CD8+ and OT-I) (figure 4B and online supplemental figure 5B). The relative deletion of T-cell populations in the mIgG2a-treated mice correlated with the amount of hOX40 surface expression seen following activation in vitro (figure 1C) as the most significant difference was seen in the Treg population followed by CD4+ effectors. The CD8:Treg ratio was unchanged following treatment with the mIgG1 mAb; however, SAP 25–29 mIgG2a produced a significant increase in the CD8:Treg ratio a with a trend towards an increase also seen for SAP 15–3 mIgG2a and SAP 9 mIgG2a (figure 4B). Interestingly, SAP 9 mIgG2a also showed a decrease in inter-sample variability when looking at OT-I cell numbers and a trend towards a reduction in CD8+ T-cell numbers, indicating superior deletion capacity compared with other mAb. Therefore, we assessed the ability of SAP 9 to delete human Tregs in vivo. Unactivated human Treg do not typically express appreciable OX40 levels (figure 1D,^34^), so a NOD/Shi-scid/IL-2Rγ^null^ (NOG): PBMC transfer model was used whereby hPBMCs are first activated through a xenoresponsive graft versus host response before being transferred into a new NOD scid gamma (NSG) recipient mouse where deletion could then be assessed. The transferred Treg upregulate hOX40 to levels similar to those observed within tumors and significantly above the levels of CD8+ T cells (online supplemental figure 5B). Using this approach, SAP 9 hIgG1 was shown to be capable of deleting human Tregs at least as well as the clinically-approved Treg deleting anti-CTLA-4 mAb Yervoy (figure 4D); thereby augmenting the CD8:Treg ratio (figure 4E). Furthermore, when using a humanized version of SAP 9 hIgG1, significant depletion of human Tregs was observed (figure 4F), alongside a significant improvement in the CD8:Treg ratio (figure 4G), unlike CAMPATH-1 which deleted all T cells. These data indicate that with the correct isotype and significant expression of hOX40, anti-hOX40 antibodies are capable of specifically depleting Tregs and improving CD8:Treg ratios in vivo.

10.1136/jitc-2020-001557.supp7 Supplementary data

### Extent of isotype activity correlates with OX40 domain specificity

**Figure 5:**
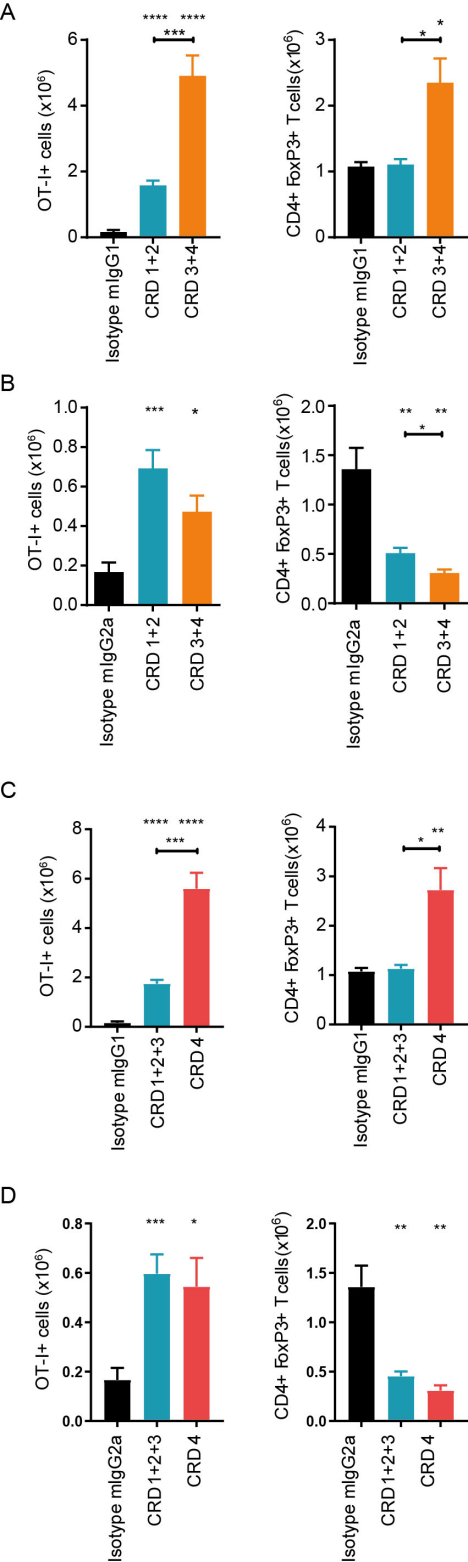
Anti-hOX40 mAb agonism and depleting activity are related to domain binding. Model used as in figure 4A and B with spleens harvested on day 4 post treatment. (A) Analysis of OT-I cell numbers (left panels) and Treg cell numbers (right panels) in response to anti-hOX40 mIgG1 mAb grouped into proximal binders —CRD3 +4 or distal binders—CRD1+2. (B) As in A except mIgG2a mAb was used. (C) Analysis of OT-I cell numbers (left panels) and Treg cell numbers (right panels) in response to anti-hOX40 mIgG1 mAb grouped into those which can bind in the presence of ligand CRD4 versus those which cannot CRD1–3. (D) As in C, except mIgG2a mAb was used. Mean±SEM ****p<0.0001, ***p<0.001, **p<0.01, *p<0.05, Tukey’s multiple comparison test (A and B; n=6–16) (C and D; n=6–19). CRD, cysteine-rich domain; mAb, monoclonal antibody.

Throughout the data detailed above, it became apparent that the strength of deletion/agonism was associated with the domain of hOX40 bound by the various mAb. To assess this further, we grouped our results into membrane distal (CRD1+2) and membrane proximal (CRD3+4) binding mAb (figure 5A and B). We also assessed groups reflecting those able (CRD4) or unable to bind in the presence of ligand (CRD1+2+3) to see if there was a correlation with ligand competition (figure 5C and D). The strength of agonism seen with the mIgG1 mAb was highest for membrane-proximal binding mAb for both expansion of OT-I cells and Tregs (figure 5A). In contrast, with the mIgG2a isotype. only depletion of Tregs correlated with domain, again being greatest for membrane proximal mAb (figure 5B). Likewise, mAb binding outside of the ligand-binding domain (ie, CRD4) displayed the highest level of mIgG1-mediated agonism (figure 5C). Interestingly, the ability of mIgG2a mAb to deplete Tregs did not significantly correlate with binding to CRD4 (figure 5D).

### Ability of hOX40 mAb to control tumor growth

**Figure 6:**
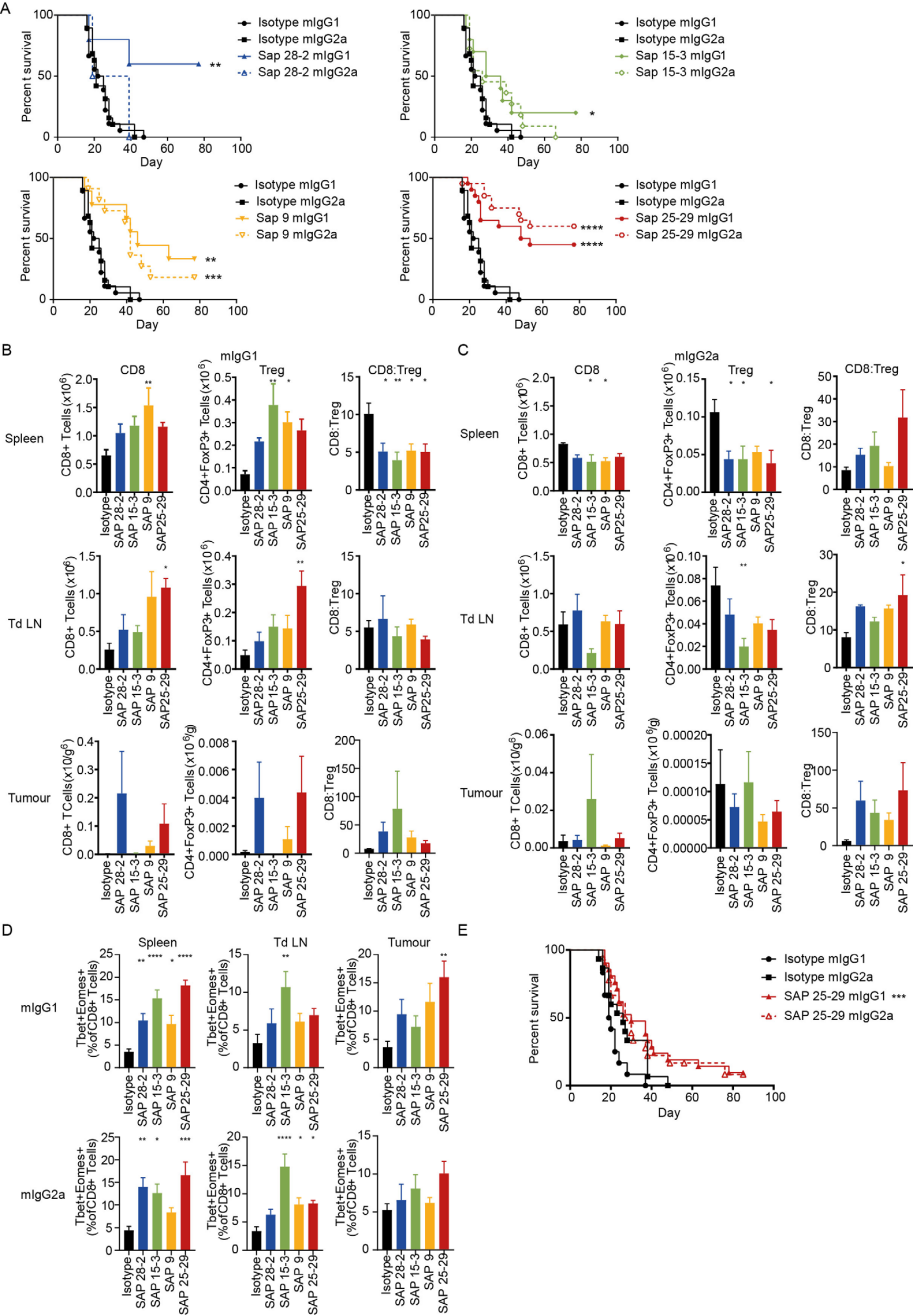
Anti-hOX40 mAb are therapeutic as both mIgG1 and mIgG2a. (A) Survival curves for mice challenged with E.G7 lymphoma cells (0.5×10^6^) and treated with 3×100 μg mAb once tumors are between 5×5 mm and 10×10 mm. Data pooled from two independent experiments (n=5 SAP 28–2, n=10 SAP 15–3 and SAP 9, n=20 isotype controls and SAP 25–29). assessment of T-cell populations in spleen (top panels), tdLN (middle panels), and tumor (bottom panels) isolated 24 hours post second mAb dose either as a mIgG1 (B) or mIgG2a (C). n=5 except for SAP 28–2 mIgG2a n=4 for all organs and SAP15–3 and SAP 25–29 mIgG1 in tumor n=3 due to tumor regression, representative of two independent experiments. (D) Analysis of T-box transcription factors and eomesodermin expression in CD8+ T cells isolated from spleen (left panels), tdLN (middle panels) and tumor (right panels) from mice treated with either mIgG1 (top row) or mIgG2a (bottom row). Data pooled from two independent experiments (n=8 SAP 28–2, all other groups n=9). (E) Survival graphs for mice challenged with MCA-205 cells (0.5×10^6^) and treated with 3×100 μg mAb once tumors were 5×5 mm. Data pooled from three independent experiments (n=12 isotype mIgG1, n=15 isotype mIgG2a, n=18 SAP 25–29 mIgG2a and n=21 SAP 25–29 mIgG2a). Mean±SEM ****p<0.0001, ***p<0.001, **p<0.01, *p<0.05, Log-rank (Mantel-Cox) for survival graphs (A and E) and Dunnett’s multiple comparison test (B to D). mAb, monoclonal antibody; tdLN, tumor-draining lymph node.

To determine the immunotherapeutic potential of the anti-hOX40 mAb in vivo in our hOX40^+/+^ KI mice, we evaluated a mAb which bound to each CRD of hOX40 as both a mIgG1 and mIgG2a isotype. hOX40^+/+^ KI mice were inoculated with E.G7-OVA lymphoma cells and subsequently treated with anti-hOX40 mAb once tumors had established. Anti-hOX40 mAb, as both mIgG1 and mIgG2a, were able to elicit tumor control, with mice eradicating established tumors in most treatment groups (figure 6A). With the exception of SAP 25–29, the mIgG1 mAb caused a higher percentage of survival than the mIgG2a mAb, although there was no obvious domain preference among the antibodies in terms of anti-tumor activity. Importantly, mice also appeared to form durable memory responses, as on re-challenge, no mice developed a secondary tumor (online supplemental figure 6A).

10.1136/jitc-2020-001557.supp8 Supplementary data

To determine the mechanism of tumor control, organs from tumor bearing mice were harvested and assessed for changes in immune inflitrate. Consistent with data from the OT-I model, the general trend seen in mIgG1-treated mice was expansion of T cell subsets while mIgG2a mAb caused T-cell depletion (figure 6B and C and online supplemental figure 6B and C). This effect was most prominent in the spleen but also observed within the tumor-draining lymph node (tdLN; CD4+ and Tregs). Cell numbers recovered from tumors were very small therefore it was difficult to ascertain clear trends within the tumor-infiltrating lymphocytes (TILs) (figure 6B and C). A high CD8:Treg within human tumors is associated with prolonged survival^35^ and depletion of Tregs allows for anti-tumor immunity and rejection.^36 37^ Our results show that mice treated with mIgG2a anti-hOX40 tended to increase the CD8:Treg ratio within the spleen, tdLN, and tumor (figure 6C). In mIgG1-treated mice, while the spleen showed a significant reduction in the CD8:Treg ratio, in the tumor, an increased trend was observed (figure 6B). It is unclear whether this discrepancy between locations occurs as a result of different response kinetics, with priming occurring in lymphoid organs and subsequent recruitment to the tumor or perhaps direct priming within the tumor microenvironment (TME) whereby the greater antigen density leads to a more rapid response and hence increase in CD8:Treg. A domain trend could also be seen within some of the T-cell subsets; OT-I depletion in the spleen and CD4+/Treg expansion within the tdLN associated with membrane proximal domains (figure 6B and C and online supplemental figure 6B and C).

The T-box transcription factors (T-bet) and eomesodermin (Eomes) cooperate to promote cytotoxic lymphocyte formation, which correlates with the upregulation of perforin and granzyme B in antigen-specific cells,^26 38 39^ as well as sustaining memory phenotypes.^40^ Thus expression of T-bet and Eomes was examined in T cells within the spleen, tdLN and tumor of anti-hOX40-treated mice to better understand the mechanisms involved. Both isotypes increased the T-bet+Eomes+double positive cells within the CD8+ populations (figure 6D); however, in CD4+ T cells, only mIgG1 increased this population and only in splenic populations (online supplemental figure 7A). A domain trend was also broadly observed in CD8+ populations from mIgG1-treated mice, most obviously within the spleen and tumor. CD8+ T cells within the spleen and tumor also saw an increase in granzyme B producing cells in both mIgG1-treated and mIgG2a-treated mice (online supplemental figure 7B). As with T-bet+ Eomes+ populations, CD4+ GzmB+ cells were not significantly increased with either isotype (online supplemental figure 7C), thus suggesting that, in this model, we do not see a significant impact on cytotoxic CD4+ T cells. However, these results do show that all anti-hOX40 mAb, irrespective of isotype or domain-binding region, are able to produce functional effector CD8+ T cells, as well as CD8+ T cells expressing transcription factors important for effector and memory cell formation within a tumor environment.

10.1136/jitc-2020-001557.supp9 Supplementary data

To determine if the ability of these mAb to evoke tumor control was consistent across tumor models we used the subcutaneous MCA-205 sarcoma. Using SAP 25–29 as a paradigm, we again saw both isotypes providing tumor control, although more limited in comparison to the E.G7 tumor model and with no isotype preference (figure 6E).

## Discussion

In the current study, we generated and characterized a panel of mAb-targeting hOX40. Their ability to bind throughout the four different hOX40 CRDs and expression as both mIgG1 and mIgG2a isotypes allowed investigation of the effects of both isotype and domain binding on agonistic and therapeutic potential in a newly developed hOX40 KI mouse model.

The hOX40 KI mouse model displayed an expression pattern of hOX40 largely reflecting that seen on healthy hPBMCs and samples from ovarian cancer patients with a hierarchy of expression of Treg>CD4+>CD8+. Nevertheless, differences were observed such that higher levels of hOX40 were seen on peripheral CD8+ T cells in the homozygous KI mouse than would be expected on hPBMC. Additionally, constitutive expression of hOX40 was observed on peripheral Tregs in the mice, in contrast to negligible levels on resting hPBMCs. However, this expression pattern reflects that observed on Treg in TILs isolated from cancer patients and so appears a reasonable model for studies in oncology.

In the hOX40 KI mouse, mOX40L not hOX40L is present which may have led to immune defects due to the absence of OX40L:OX40 interaction. However, no overt differences in immune development or homeostasis were observed in the homozygous hOX40 KI mice. Furthermore, mOX40L has been shown to make similar contacts to hOX40 as hOX40L,^21^ suggesting that the OX40L:OX40 signals would be maintained in our model. It also indicates that the model is suitable to address any influences of ligand binding on the activity of the mAb panel. Only mAb binding to the most membrane proximal domain (CRD4), were able to bind in the presence of the natural ligand OX40L (CD252), raising the possibility that certain effector functions could be influenced by concurrent ligand binding for the CRD4-binding mAb. Although not studied directly, no overt effects appeared to be driven by the presence or absence of OX40L binding; that is, all antibodies regardless of ligand blocking were able to elicit function in an OT-I transfer model and in tumor models.

It is well known that isotype helps dictate mAb effector function due in part to differences in FcγR interactions, and so isotype choice is important for delivering therapeutic efficacy according to the mAb mechanism of action.^24 41 42^ For TNFR family members, mIgG1 antibodies have been agonistic with engagement of FcγRII and mIgG2a being either inhibitory or with limited agonistic effects yet capable of activatory FcγR-mediated target cell depletion.^12 18 24 25^ Our hPBMC proliferation data added to this evidence, with hOX40 mIgG2a mAb resulting in a decrease in the percentage of proliferating T cells whereas mIgG1 counterparts evoked increases in T-cell proliferation. However, this was not reflected in our hOX40^+/−^ OT-I transfer studies in hOX40^+/+^ KI mice, where both anti-hOX40 mAb isotypes were able to cause strong expansion of OT-I cells. However, when hOX40^+/−^ OT-I cells were transferred into WT mice, the mIgG2a mAb were no longer able to elicit expansion of OT-I cells, unlike the mIgG1 mAb, which retained this activity. Together, these results indicate that the mIgG1 mAb causes direct agonism on the hOX40^+/−^ OT-I cells, resulting in their expansion, whereas the mIgG2a mAb require hOX40 expressing non-CD8+ cells to provide expansion. Previous experiments by Ruby *et al*^43^ indicated a requirement for CD4+ T cells in the expansion of OT-I T cells via anti-mouse OX40 mAb. In those experiments in Major Histocompatibility Complex (MHC) Class II KO mice, a reduction in the resting memory population was observed, with no significant change at the peak of the primary response as we show here with the mIgG2a. There are many differences in experimental set up, including the number of OT-I cells transferred, and site of priming, as well as the isotype used. Experiments performed by Ruby *et al*^43^ used the anti-mOX40 mAb OX86 which is a Rat IgG1 (rIgG1). rIgG1 interacts with only FcγRIIb and FcγRIII, giving it a low activatory FcγR: inhibitory FcγR binding (A:I) ratio, in contrast to the mIgG2a which interacts strongly with all activatory FcγR and has a higher A:I ratio.^44^ Thus, while the mIgG2a is likely to mediate its effects through depletion, based on strong activatory FcγR interactions, the rIgG1 through its relatively greater interaction with FcγRIIb is likely to have the additional capacity of direct agonism. Hence in the MHC Class II KO mice, there is the possibility for the OX86 rIgG1 to directly agonize the transferred OT-I T cells via FcγRIIb-mediated crosslinking, unlike the ahOX40 mIgG2a when used in WT recipients, potentially explaining the disparity between the two data sets.

Enumerating the subpopulations of T cells within the spleen of hOX40^+/+^ KI mice revealed that the mIgG2a anti-hOX40 mAb uniquely caused depletion of Tregs with lower levels of depletion seen in the CD4+ effector population. In contrast, the mIgG1 anti-hOX40 mAb caused expansion of all T-cell populations, most significantly in the general CD8+ and OT-I populations. Assuming the presence of relevant effector cells and normal distribution of FcγRs in our OT-I model, it is likely that differential FcγR interactions of the different isotypes explains their disparate effects. Furthermore, the superior affinity for the activatory FcγR and resultant deletion of suppressive Treg cells seen in mice treated with mIgG2a mAb likely explain the mechanism behind the expansion of OT-I cells in the blood and spleen of hOX40^+/+^ KI but not WT mice.

Another disparity seen between the anti-hOX40 mIgG1 and mIgG2a-treated mice was the recall response to SIINFEKL peptide in the OT-I model. Considering the similar levels of OT-I cells at the peak of the primary response, it was surprising to observe such a difference in the frequency in the memory phase. A positive correlation between the frequency of OT-I cells pre-recall and the frequency of OT-I cells at the peak of the memory response highlighted the possibility that it was simply the result of the amount of cells present at the time of re-challenge, with mIgG1 mAb but not mIgG2a mAb providing signals for survival/persistence. Within the primary response the MPEC population (CD127^+^KLRG1^−^) was increased at day 18 in mice treated with mIgG1 mAb compared with those treated with mIgG2a. Those mice also generated a higher percentage of CXCR3^hi^CD43^lo^ highly proliferative cells compared with mice treated with the mIgG2a mAb. These findings, alongside those relating to the frequency of antigen-specific cells pre-rechallenge explain the disparity in the recall response comparing mIgG1 versus mIgG2a mAb. The exact mechanisms underpinning this dichotomy are not immediately clear but one possibility is that mIgG2a deplete Tregs alongside, to a lesser extent, CD4+ effectors, to influence priming. Importantly though, this higher proportion of effector cells in mIgG2a-treated mice versus highly proliferative cells in mIgG1-treated mice may explain these differences in memory but moreover provide a rationale for how both isotypes are able to cause equivalent efficacy in mouse tumor models, despite exhibiting opposing mechanisms of action.

Analysis of affinity, on and off rates failed to reveal a correlation with activity. However, despite the limited numbers of antibodies against each individual domain, our data suggests a correlation between domain binding and strength of both mIgG2a depletion and mIgG1 agonism. Anti-hOX40 mAb, which bound to CRD4, were more potent agonists as mIgG1 when compared with mAb which bound to CRD1-3, although testing against a wider panel of antibodies would be required to strengthen this finding. This directly contrasts with our previous observations with anti-CD40 mAb^16^ where CRD1 binding mAb were more agonistic. However, for both the anti-hCD40 and anti-hOX40 mAb tested, optimal agonistic function correlated with binding outside the natural ligand binding region. This potentially suggests that the combined effects of both the ligand and the mAb in clustering the receptor are required to elicit optimal agonism. This conjecture is partially supported by recent evidence from Zhang *et al*^17^ who also demonstrated strong agonistic function with a CRD4-binding anti-mOX40 mAb. Those authors also documented equivalent agonistic activity with a CRD2-binding, ligand-blocking anti-mOX40 mAb indicating the fine epitope is also important, as we reported previously for anti-CD40.^16^

The ability of TNFR targeting mAb to cause depletion of intratumoral Tregs has been clearly demonstrated in recent years,^8 12 45 46^ both increasing their therapeutic possibilities but confusing potential mechanisms of action. To assess, in a more translational setting, the depleting ability of our anti-hOX40 mAb, we performed depletion experiments in NSG mice engrafted with human target cells. In these experiments, hOX40 and other TNFR family members become upregulated on the activated Treg (online supplemental figure 5B^13^), enabling them to serve as targets akin to those seen in tumor samples. When compared with the clinically relevant Treg depletor Yervoy,^47^ SAP 9 hIgG1 was not only as strong a Treg depletor but also generated a higher CD8:Treg ratio, indicating the therapeutic potential of this anti-hOX40 mAb. Importantly, deletion of Treg was specific even though all T-cell subsets had expanded and were activated through the NSG passage.

Having established the agonistic and depletory capacity of our mAb, it was then perhaps slightly surprising for both isotypes to act therapeutically to a similar extent in tumor models but likely reflects their relative propensity to elicit different effector functions in the TME. The latter is known to have profound effects on therapeutic efficacy.^48^ A tumor with a high infiltrate of cells expressing activatory FcγR such as NK cells and macrophages, is likely to be more responsive to a mIgG2a mAb and depletion of detrimental target cells than a T-cell-agonizing mIgG1 mAb. Conversely, if the inhibitory FcγRIIB is more prevalent, mIgG1-mediated agonism may be more prominent. Lymphoid organs outside the tumor may also be relevant. In the E.G7-OVA model, although the magnitude of the effect differed, the mIgG1 mAb largely caused T-cell expansion and the mIgG2a depletion in spleen and draining lymph nodes. It seems likely therefore that these intrinsic differences underpin the relevant mechanism of action in each case. Accordingly, mIgG2a reagents likely achieve therapeutic effects through depleting Tregs, releasing T-cell effector responses whereas mIgG1 expand T-cell numbers and hence increase cytotoxic effectors within the tumor site. Despite the disparity in recall responses in the OT-I model (mIgG1 >>mIgG2 a), in the E.G7-OVA model both isotypes were able to elicit tumor control and generate memory sufficient to prevent tumor growth on rechallenge. This suggests that other factors must be operational in the presence of tumor. One possibility is that the threshold for memory is relatively low in the presence of an immunogeneic tumor and that the limited level of memory recall seen following treatment with the mIgG2a is sufficient. Alternatively, it may indicate that the mIgG2a mechanism of action, deleting Tregs, allows the expansion of otherwise cryptic epitopes allowing T-cell control as was previously described in the CT26 model.^49^

In recent years, evidence for cytotoxic CD4+ T cells in tumor eradication has been reported.^50 51^ Through the use of both adoptive transfer models and the B16 F10 melanoma model, Qui *et al*^51^ showed that OX40 mAb stimulation increased GzmB production in CD4+ T cells and that dual co-stimulation with 4-1BB could expand these cells. In our E.G7 model, however, we failed to see consistent increases in either CD4+GzmB+T cells or CD4+Eomes+T-bet+T cells in response to anti-OX40 mAb (online supplemental figure 7A–C) regardless of isotype used. This potentially reflects the differences in how GzmB was measured in the two tumor models as in the B16 F10 model, isolated cells were stimulated for 24 hours with anti-CD3 mAb prior to GzmB detection. As we measured GzmB directly ex vivo, we cannot rule out that on restimulation we would also have revealed CD4+ cytotoxic potential in response to OX40 stimulation.

In summary, our findings show that immunomodulatory mAb directed against hOX40 can harness multiple mechanisms of action to elicit tumor control. They also show that these mechanisms can be modulated dependent on the choice of isotype and domain-binding region. Targeting the membrane proximal domains appears optimal for both deletion and agonism; with the latter strongly driven by isotypes with low A:I ratios such as mIgG1. Lowering the A:I ratio can be attained in many different ways, for example, by increasing the affinity for FcγRIIB, which has been shown to mediate more effective agonism for anti-CD40 mAb^52^ and a recent paper showed that this may also be true for OX40 using a clinically relevant antibody^53^ in vitro but as yet it is unclear whether this will be true in vivo for anti-hOX40 mAb. These findings have implications for the design of the next generation of anti-hOX40 mAb for the clinic. Current reagents typically display an unmodified hIgG1 isotype and although safe have not delivered strong anti-tumor effects.^6^ These hIgG1 reagents would be expected to deliver the Treg-deleting function indicated here but to date this activity has not been shown clearly in patients. Furthermore, their deletion may not be sufficient to elicit tumor regression in most human cancers, unlike the mouse models shown here. Therefore, mAb with appropriate isotypes and further engineering (hIgG2B,^16 54^ SELF^52 55^ and V11^52 56^) to elicit potent T-cell agonism may be warranted for further investigation for use both as a monotherapy but more importantly in combination.

## Methods and materials

### Human samples

PBMCs were obtained from healthy adult volunteers from either Southampton National Blood Service, UK, or Hallands Hospital Halmstad, Sweden. For NSG reconstitution experiments performed in Southampton, hPBMCs were purchased from STEMCELL Technologies.

### Mice

C57BL/6 mice and OT-I transgenic mice were obtained from Charles River Laboratories. NSG mice were purchased from Jackson Laboratories. hOX40 KI mice were generated by Ozgene. hOX40/OT-I mice were generated in-house. For all experiments, young adult mice were sex-matched and age-matched and randomly assigned to experimental groups. Experiments were not blinded.

### Antibody production and labeling

Anti-hOX40 mAb were produced and labeled using standard techniques as detailed in online supplemental material 1. Cetuximab was a kind gift from Thomas Valerius and Campath-1 was a kind gift from Geoff Hale.

10.1136/jitc-2020-001557.supp3 Supplementary data

### Humanization of SAP 9

The variable regions of SAP 9 heavy and light chains were sequenced from the hybridoma by PCR. The sequence was humanized using Macromoltek’s proprietary humanisation algorithms. A generic antibody signal peptide sequence was then added to the humanized variable region sequences and the amino acid sequences converted into nucleotide sequences using https://www.bioinformatics.org/sms2/rev_trans.html. Nucleotide sequences were synthesized by GeneArt and subcloned into expression vector pEE6.4 (Lonza) for expression.

Binding domain determination/blocking experiments hOX40 constructs were transiently transfected into 293 F cells before addition of 10 µg/mL anti-hOX40 mAb, binding was detected with a PE-labeled secondary anti-mouse Fc antibody (Jackson Laboratories). For blocking experiments, unlabeled antibody was added for 30 min before addition of FITC-labeled anti-hOX40 mAb. Binding was assessed using flow cytometry (see below).

### Surface plasmon resonance

A Biacore T100 upgraded to a T200 (GE Life Sciences) was used to measure interactions with hOX40. 100 nM of hOX40L-His was immobilized onto a CM5 chip (GE Healthcare) coated with an anti-His mAb. 100 nM hOX40-hFc was subsequently captured followed by the injection of anti-hOX40 mAb (15 µg/mL).

### In vitro assays

Standard hPBMC proliferation assays were performed as detailed in online supplemental material 1. For expression assays, frozen hPBMCs were thawed and rested overnight, then stimulated with plate-bound anti-CD3 (OKT3, 15 ng/mL) and soluble anti-CD28 (CD28.2, 0.5 µg/mL). Cells were harvested, stained with appropriate antibodies and assessed via flow cytometry.

Murine expression assays; standard activation was used as detailed in online supplemental material 1. Cells were stained with appropriate antibodies and analyzed by flow cytometry.

### Flow cytometry

Standard staining and analysis performed as detailed in online supplemental material 1. Antibodies used are detailed in online supplemental table 1.

10.1136/jitc-2020-001557.supp2 Supplementary data

### OT-I adoptive transfer

A total of 1×10^5^ hOX40 KI^+/−^ OT-I cells were injected intravenously into hOX40 KI^+/+^ or WT C57BL/6 mice. Twenty-four hours later 5 mg ovalbumin (Sigma) and 100 µg anti-hOX40 or isotype control were given intraperitoneally (i.p.). Deletion was determined by harvesting spleens day 4 post i.p. injection. OT-I kinetics were monitored in the blood through SIINFEKL tetramer staining and mice were rechallenged between 6 and 10 weeks later with 30 nM SIINFEKL intravenously. Based on preliminary experiments, n=3 was determined as sufficient to see a p<0.05 for OT-I expansion. Mice with SIINFEKL tetramer responses less than 1% of CD8+ lymphocytes at the peak of the response were excluded due to the likelihood that the OT-I transfer had failed since isotype controls peak at an average 5.2%±0.58 SEM (mIgG1) and 4.65%±0.65 SEM (mIgG2a) in blood and 3.8%±0.79 SEM (mIgG1) and 3.1%±0.89 on Day 4 in spleens. One mouse was excluded from figure 4B groups SAP-28, 15–3 and 9 based on this criteria.

### Treg cell depletion in reconstituted NOG/SCID mice

PBMC-NOG/severe combined immune-deficient (SCID) mice (primary human xenograft model) were generated by injecting NOG or NSG mice with 1–1.5×10^7^ PBMC isolated using Ficoll-Paque PLUS. Approximately 2 weeks after reconstitution of NOG or NSG mice with hPBMCs, spleens were harvested. Splenocytes 10×10^6^ were then injected into the peritoneal cavity of naive SCID or NSG mice 1 hour prior to injection with 10 mg/kg of depleting mAb or isotype control mAb. The peritoneal fluid was collected after 24 hours and human T-cell subsets were identified by flow cytometry.

### Tumor models

E.G7-OVA and MCA-205 tumor models: 5×10^5^ tumor cells were injected subcutaneously into the flank of mice. Based on preliminary experiments, n=5 was determined as sufficient to see a p<0.05 for tumor therapy. Groups of eight (eg, 7-OVA) or six (MCA-205) mice were set up to ensure that at treatment there would be a minimum of 5 per group with established tumors with comparable size (between 5×5 mm and 8×8 mm). Mice were then ranked according to tumor size and assigned to treatments groups so that average tumor size per group was similar prior to treatment. This ensured mixed treatment groups within cages to reduce influence of housing on treatment effect. Established tumors were treated with 3×100 µg anti-hOX40 mAb or isotype i.p. every other day. For phenotyping experiments, organs were harvested day 4 post final injection. Tumors were digested using 0.5 units of liberase TL (Roche) and cells analyzed via flow cytometry. For survival experiments, tumor size mice were culled once they reached a terminal size (eg,7: 20×20 mm, MCA-205: 15×15 mm). Mice which eradicated tumor after treatment were rechallenged with 5×10^5^ tumor cells subcutaneously into the flank.

### Statistics

All results show mean±SEM. One-way Anova with multiple comparisons (Dunnett’s, Tukey’s or Sidak’s as stated in legend) or Mann-Whitney tests were used as stated in legends performed using GraphPad Prism. Survival curves evaluated using a Log-rank (Mantel-Cox) test. Significance is shown relative to isotype control unless bar is shown. Where indicated, ns=not significant, *p≤0.05, **p≤0.01, ***p≤0.001, ****p≤0.0001.
