# Supplementary material for: Domain binding and isotype dictate the activity of anti-human OX40 antibodies
Source: J Immunother Cancer. 2020 Dec 21;8(2):e001557. doi: 10.1136/jitc-2020-001557 (PMC7754644; doi:10.1136/jitc-2020-001557)
Supplement: Supplementary data [file jitc-2020-001557supp001.pdf]

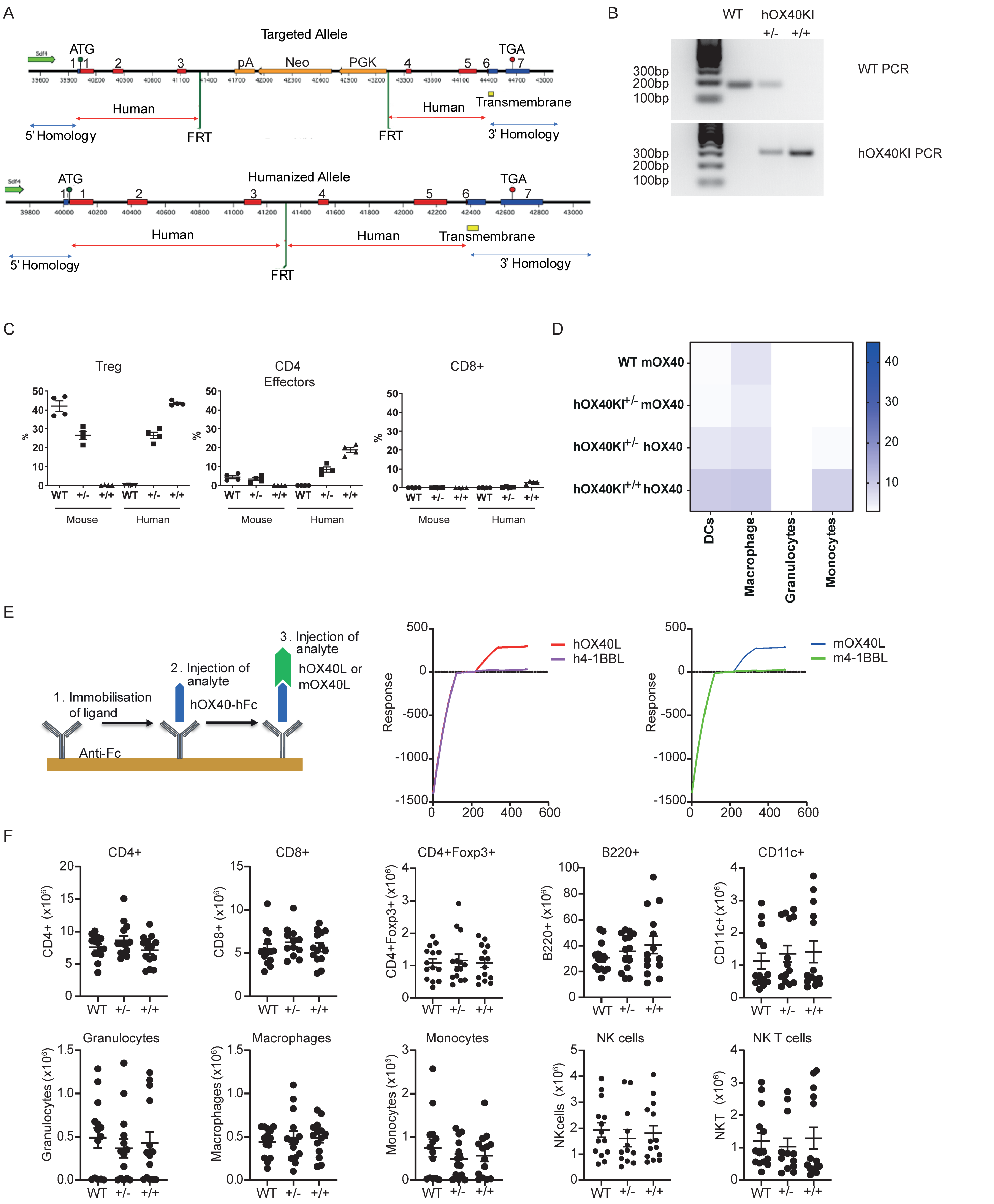

Supplementary Figure 1. hOX40 expression in hOX40KI mice is dose dependent. A. Schematic of hOX40 chimeric receptor construct used to generate the hOX40KI mice. B. PCR showing the genotyping of WT, hOX40KI$^{+/-}$ and hOX40KI$^{+/+}$ mice. C. Expression of mouse and human OX40 on Treg (left panel), CD4 effectors (middle panel) and CD8 T cells (right panel) isolated from WT, hOX40KI$^{+/-}$ and hOX40KI$^{+/+}$ n=4. D. Heat map showing expression levels on myeloid populations. E. SPR analysis of OX40 L binding to hOX40. Left panel shows schematic, h4-1BBL and m4-1BBL were used as negative controls. Middle panel shows hOX40L (red) and h4-1BBL (purple) binding and right panel shows mOX40L (blue) and m4-1BBL (green) F. Lymphocyte and Myeloid populations in WT, hOX-40KI$^{+/-}$ and hOX40KI$^{+/+}$ mice (6-10 weeks age, n=14).
