# Supplementary figures and images for: Domain binding and isotype dictate the activity of anti-human OX40 antibodies
Source: J Immunother Cancer. 2020 Dec 21;8(2):e001557. doi: 10.1136/jitc-2020-001557 (PMC7754644; doi:10.1136/jitc-2020-001557)

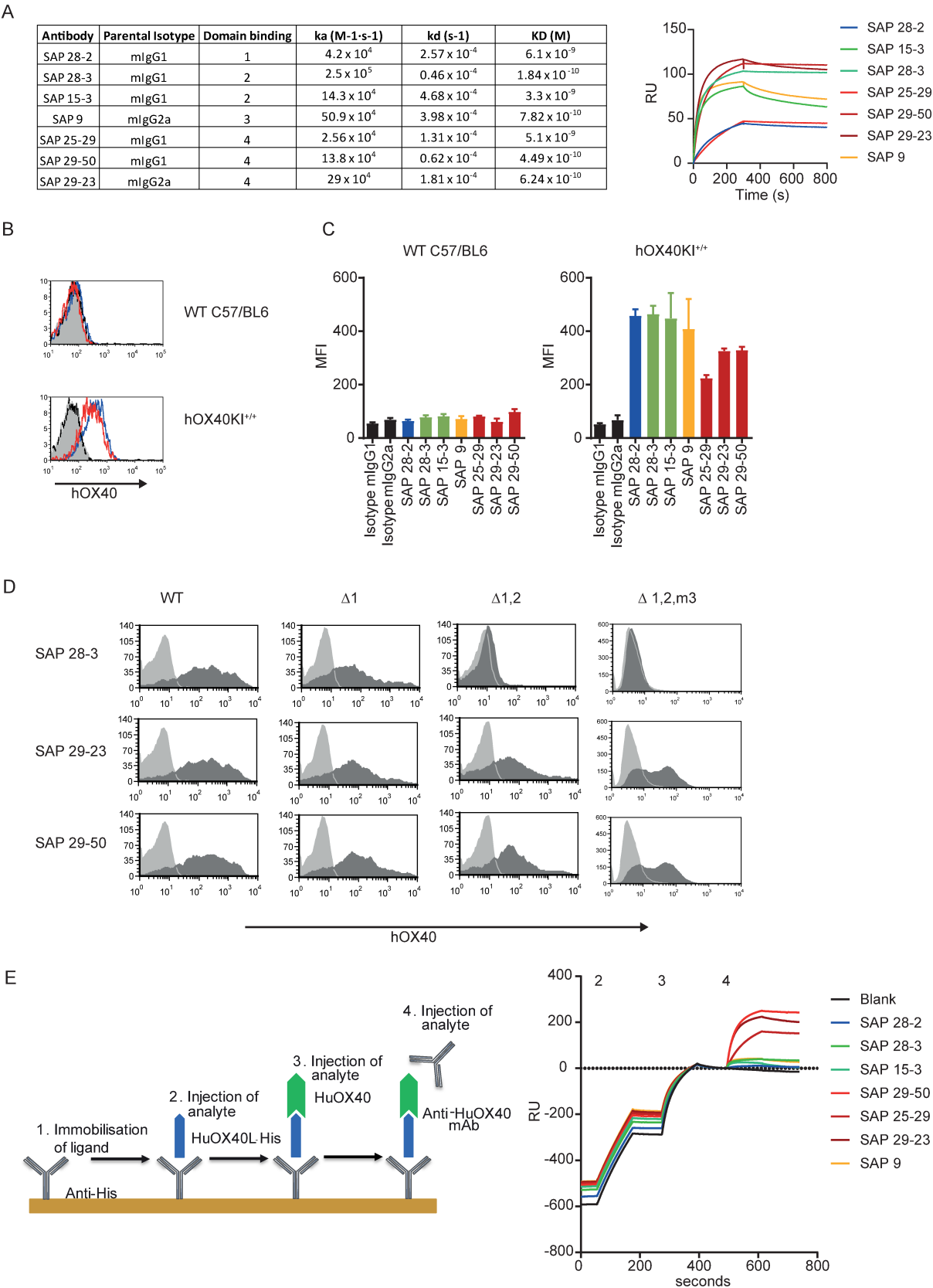

Supplement: Supplementary data [file jitc-2020-001557supp004.pdf]
