# Supplementary material for: Domain binding and isotype dictate the activity of anti-human OX40 antibodies
Source: J Immunother Cancer. 2020 Dec 21;8(2):e001557. doi: 10.1136/jitc-2020-001557 (PMC7754644; doi:10.1136/jitc-2020-001557)
Supplement: Supplementary data [file jitc-2020-001557supp005.pdf]

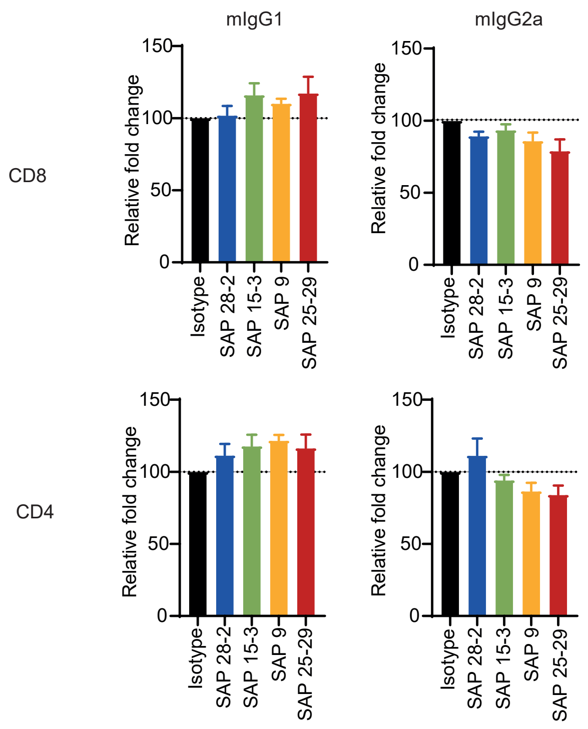

Supplementary Figure 3. anti-hOX40mAb mIgG1 promotes proliferation whilst mIgG2a reduces proliferation. hOX40KI$^{+/+}$ splenocytes were activated with αCD3 (1μg/ml) and αhOX40 mAb (10μg/ml) for 72 hours and then CFSE dilution analysed. Data presented as fold change over isotype contol, n=5.
