# Supplementary material for: Domain binding and isotype dictate the activity of anti-human OX40 antibodies
Source: J Immunother Cancer. 2020 Dec 21;8(2):e001557. doi: 10.1136/jitc-2020-001557 (PMC7754644; doi:10.1136/jitc-2020-001557)
Supplement: Supplementary data [file jitc-2020-001557supp006.pdf]

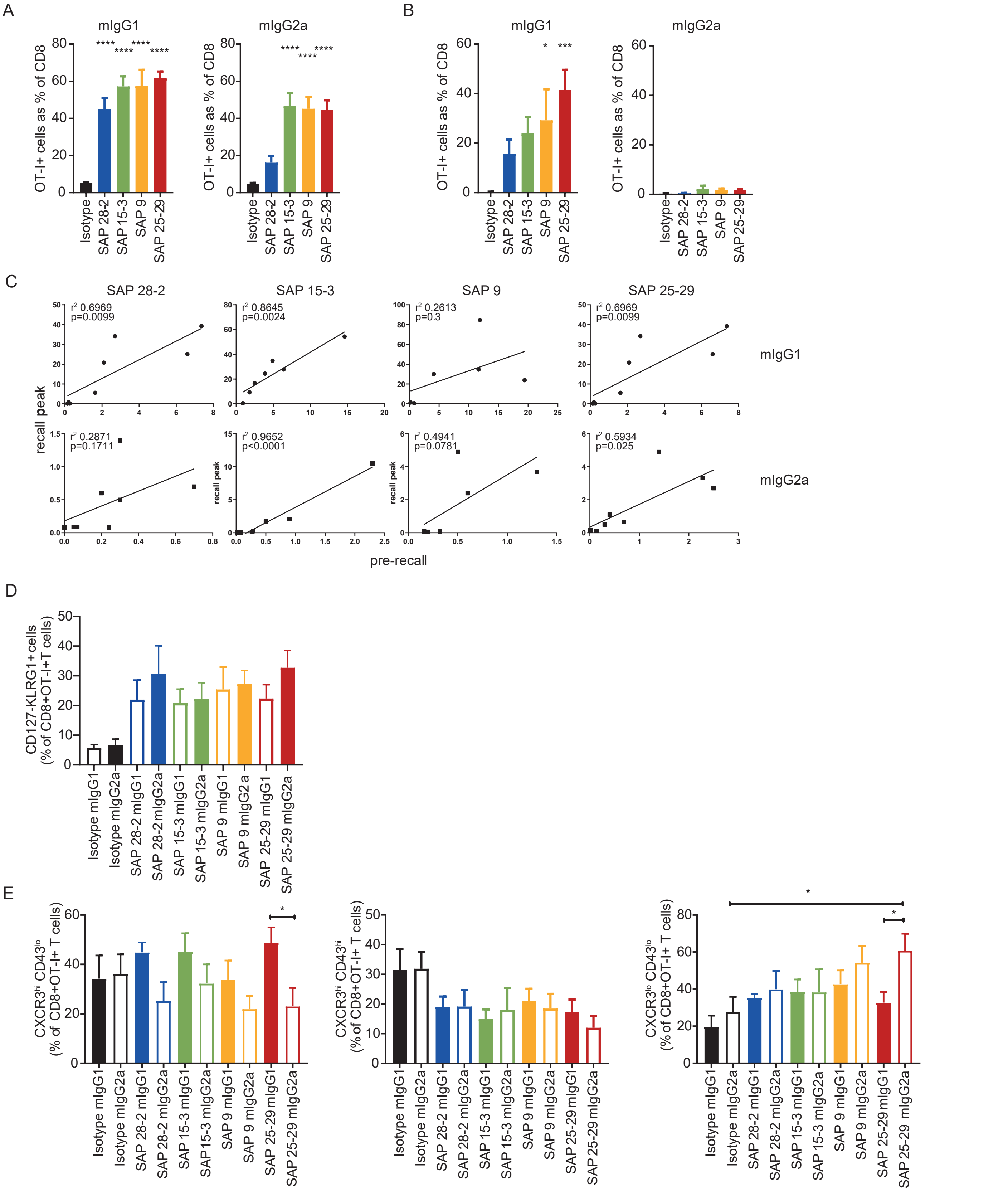


Supplemental Figure 4. anti-hOX40 mIgG1 act agonistically *in vivo*. A. Primary peak response of OT-I expansion in blood to anti-hOX40 mIgG1 (left panel) or mIgG2a (right panel) (n=6-8, pooled from 2 independent experiments). B. Recall peak following SIINFEKL rechallenge of mice previously treated with anti-hOX40 mIgG1 (left panel) or mIgG2a (right panel) (n=6-8, pooled from two independent experiments). C. Correlation graphs between recall response and pre-recall OT-I levels in anti-hOX40 mIgG1 (top row) and mIgG2a (bottom row) treated mice. n=6-8, pooled from two independent experiments. D. Analysis of OT-I SLECs CD127-KLRG1+ in the blood at D18 (n=8). E. CXCR3 and CD43 analysis of OT-I in the blood pre-rechallenge with SIINFEKL peptide (n=7-8 pooled from 2 independent experiments). CXCR3hiCD43lo (left panel), CXCR3hiCD43hi (middle panel) and CXCR3loCD43lo (right panel). ****p<0.0001, *** p< 0.001, ** p<0.01, *p<0.05 A and B - Dunnett's and E - Sidak's test.
