# Supplementary material for: Domain binding and isotype dictate the activity of anti-human OX40 antibodies
Source: J Immunother Cancer. 2020 Dec 21;8(2):e001557. doi: 10.1136/jitc-2020-001557 (PMC7754644; doi:10.1136/jitc-2020-001557)
Supplement: Supplementary data [file jitc-2020-001557supp007.pdf]

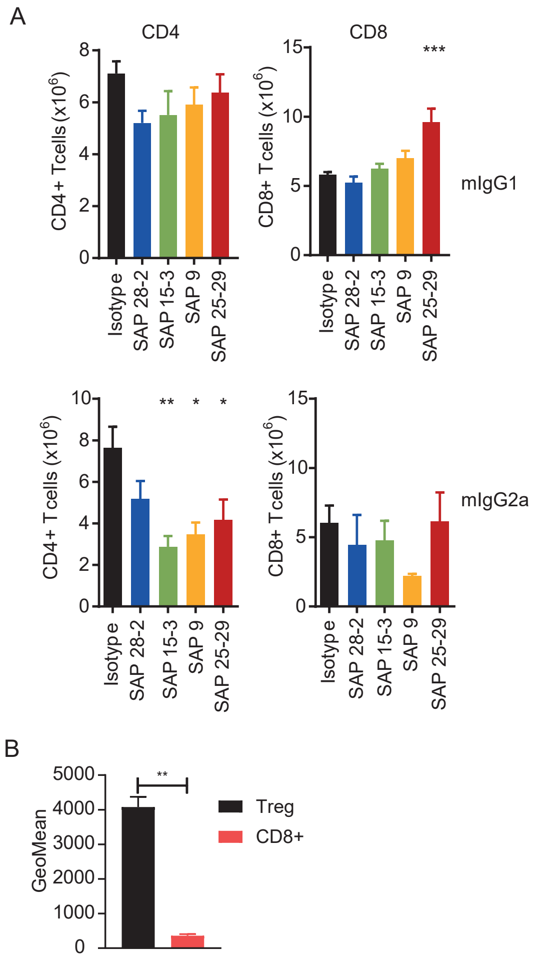

Supplemental Figure 5. Isotype of anti-hOX40 mAb influences effector function. A. CD4 and CD8 T cell numbers assessed on Day 4 in splenocytes isolated from hOX40KI$^{+/+}$ mice receiving 1x10^5 hOX40KI$^{+/-}$OT-I followed by 100μg anti-hOX40 mAb as either a mIgG1 or mIgG2a (n=7-8, pooled from 2 independent experiments). ** $p<0.01$, * $p<0.05$ Dunnett's test. B. hOX40 expression on Treg and CD8+ T cells isolated from NOG mice 2 weeks post injection of hPBMCs (n=5, representative of 2 independent experiments). ** $p<0.01$ Mann Whitney U test.
