# Supplementary material for: Domain binding and isotype dictate the activity of anti-human OX40 antibodies
Source: J Immunother Cancer. 2020 Dec 21;8(2):e001557. doi: 10.1136/jitc-2020-001557 (PMC7754644; doi:10.1136/jitc-2020-001557)
Supplement: Supplementary data [file jitc-2020-001557supp008.pdf]

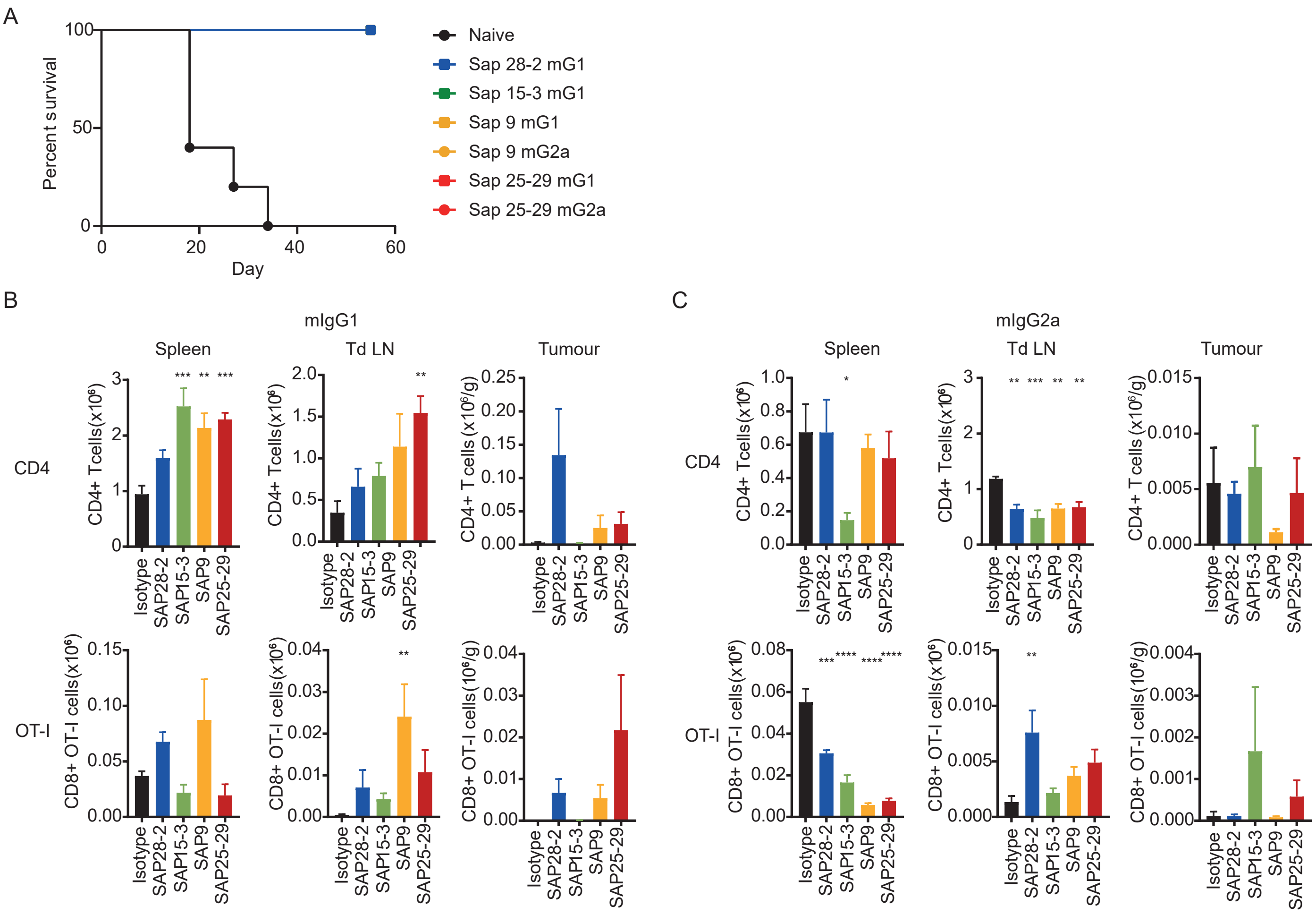

Supplementary Figure 6. hOX40mAb are therapeutic as both mIgG1 and mIgG2a. A. Survival graphs of mice rechallenged with E.G7 lymphoma cells shown alongside naive recipients. n=1-5 representative of 2 independent experiments. B and C. Assessment of CD4+ (top rows) and CD8+OT-I (bottom rows) T cell populations in Spleen (left panels), Tumour draining lymph node (middle panels), and Tumour (right panels) isolated 24 hours post second mAb dose either as a mIgG1 (B) or mIgG2a (C). n=3-5, representative of 2 independent experiments. ****p<0.0001, *** p< 0.001 ** p<0.01 * p<0.05 Log-rank (Mantel-Cox) for survival graphs A and Dunnett's multiple comparison test for B-C.
