# Supplementary material for: Domain binding and isotype dictate the activity of anti-human OX40 antibodies
Source: J Immunother Cancer. 2020 Dec 21;8(2):e001557. doi: 10.1136/jitc-2020-001557 (PMC7754644; doi:10.1136/jitc-2020-001557)
Supplement: Supplementary data [file jitc-2020-001557supp009.pdf]

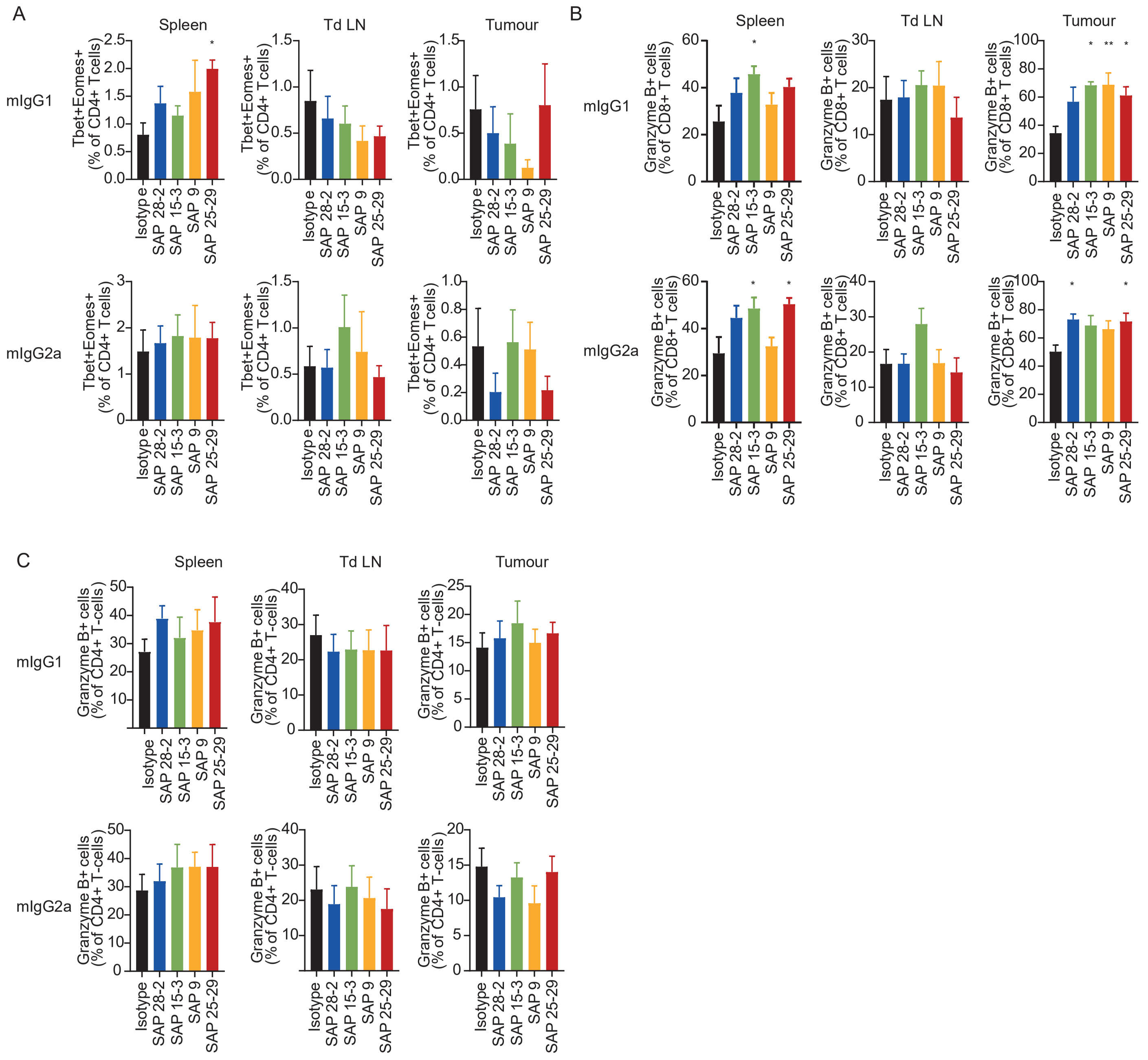

Supplementary Figure 7. Both mIgG1 and mIgG2a promote the induction of Granzyme B+ CD8+ T cells but not CD4+ T cells. A-C. Assessment of CD4+Tbet+Eomes+ (A), CD8+ GranzymeB+ (B) and CD4+GranzymeB+ (C) populations isolated from Spleen (left panels), Tumour draining lymph node (middle panels) and Tumour (right panels) from mice treated with either mIgG1 (top row) or mIgG2a (bottom row). Data pooled from 2 independent experiments (n=8-9). ****p<0.0001, *** p< 0.001 ** p<0.01 * p<0.05 Dunnett's multiple comparison test.
