# Supplementary material for: Domain binding and isotype dictate the activity of anti-human OX40 antibodies
Source: J Immunother Cancer. 2020 Dec 21;8(2):e001557. doi: 10.1136/jitc-2020-001557 (PMC7754644; doi:10.1136/jitc-2020-001557)
Supplement: Supplementary data [file jitc-2020-001557supp003.pdf]

BMJ Publishing Group Limited (BMJ) disclaims all liability and responsibility arising from any reliance placed on this supplemental material which has been supplied by the author(s)

Supplementary Methods

PCR genotyping of mice

hOX40 KI was detected using the following primers F – AGTGCCCACGCTTCCTGAGGA and R - CTTGAGGATGCCAGAGGAGGC which give a 290bp product. mOX40 was detected using the following primers F -TCTCCACCCACCTTGGTGACT and R – GCCAGCAGGACAGTCAAGGA which give a 174bp product.

Antibody Production and labelling

Anti-hOX40 mAb were created using conventional hybridoma technology [49]**.** Isotype switching was carried out as previously described [50] and mAb purified either using a protein-G or a protein-A column. All preparations were deemed endotoxin low (<1ng/mg protein). Anti-hOX40 mAb were FITC (Sigma) labelled at a ratio of 1:10 FITC:mAb and purified using a desalting column (GE Healthcare).

Surface Plasmon Resonance

A Biacore T100 upgraded to a T200 (GE Life Sciences) was used to measure interactions with hOX40. For affinity determination 1μg/ml of hOX40-hFc was immobilised onto a CM5 chip (GE Healthcare) and a range of concentrations (0-500nM) of anti-hOX40 mAb was injected over the chip. For ligand binding to hOX40; 1μg/ml of hOX40-hFc was immobilised onto a CM5 chip coated with an anti-Fc antibody and hOX40L, mOX40L, h4-1BBL or m4-1BBL (15μg/ml) was injected over the chip.

In vitro assays

hPBMC proliferation assays: hPBMCs were isolated using lymphoprep separation. For proliferation assays cells were labelled with 1μM CFSE immediately following isolation, cultured at high density ($1.5x10^7$ cells/ml) for 2 days at 37°C, then plated at $1x10^5$ cells/well. Cells were stimulated with soluble anti-CD3 (OKT3, 5ng/ml) and anti-hOX40 mAb (5μg/ml). Cells were

stained with appropriate antibodies and CFSE dilution assessed via flow cytometry.

Murine expression assays; splenocytes were isolated using a 100μm strainer and red cell lysed using ACK lysis buffer. Cells were plated at $2x10^5$ cells/well and stimulated with soluble anti-CD3 (145-2C11, 0.1μg/ml) and anti-CD28 (37.51, 1μg/ml). Cells were stained with appropriate antibodies and analysed by flow cytometry.

Murine proliferation assays; splenocytes were isolated using a 100μm strainer and red cell lysed using ACK lysis buffer. Cells were labelled with 5μM CFSE, plated at $1x10^5$ cells/well and stimulated with platebound anti-CD3 (145-2C11, 1μg/ml) and anti-OX40 mAb (10μg/ml). Cells were incubated for 72 hours, stained with appropriate antibodies and analysed for CFSE dilution by flow cytometry.

Flow cytometry

Flow cytometry antibodies are listed in Supplementary Table 1. Intracellular staining was performed using Foxp3 staining buffer kit (ThermoFisher-eBioscience) according to the manufacturer’s protocol. Cells from ovarian cancer patients were incubated with 10mg/ml KIOVIG (Baxalta) prior to staining with relevant antibodies. All flow cytometry experiments were performed using either a FACSCalibur, FACSCanto, FACSAria or FACSVerse machine (all BD Bioscience). Data analysed using Cellquest Pro, FACSDiva (V6.1.2), FlowJo (all BD Bioscience) or FCS Express (V.3) software (De Novo Software).
