# Supplementary material for: Domain binding and isotype dictate the activity of anti-human OX40 antibodies
Source: J Immunother Cancer. 2020 Dec 21;8(2):e001557. doi: 10.1136/jitc-2020-001557 (PMC7754644; doi:10.1136/jitc-2020-001557)
Supplement: Supplementary data [file jitc-2020-001557supp002.pdf]

Target	Clone	Company
mOX40	OX-86	ThermoFisher-eBioscience
mCD8a	53-6.7	ThermoFisher-eBioscience
mCD4	GK1.5	ThermoFisher-eBioscience
mCD3	145-2C11	ThermoFisher-eBioscience
mNK1.1	PK136	ThermoFisher-eBioscience
mFoxp3	FJK-16s	ThermoFisher-eBioscience
mB220	RA3-6B2	ThermoFisher-eBioscience
mCD11c	N418	ThermoFisher-eBioscience
mF4/80	BM8	ThermoFisher-eBioscience
mCD11b	M1/70.15	ThermoFisher-eBioscience
mCD62L	MEL-14	ThermoFisher-eBioscience
mCD44	IM7	ThermoFisher-eBioscience
mKLRG1	2F1	ThermoFisher-eBioscience
mCD127	A7R34	ThermoFisher-eBioscience
mCXCR3	CXCR3-173	ThermoFisher-eBioscience
mCD45.2	104	ThermoFisher-eBioscience
mT-bet	eBio4B10	ThermoFisher-eBioscience
mEOMES	DAN11MAG	ThermoFisher-eBioscience
mCD43 (activation glycoform)	1B11	BioLegend
mLy6C	HK1.4	BioLegend
mLy6G	1A8	BioLegend
h/m Granzyme B	GB11	ThermoFisher-Invitrogen
H-2K^{b}/SIINFEKL tetramer		In house
hCD4 (healthy samples)	RPA-T4	ThermoFisher-eBioscience
hCD8 (healthy samples)	SK1	ThermoFisher-eBioscience
hOX40 (healthy samples)	ACT35	ThermoFisher-eBioscience
hCD127 (healthy samples)	eBioRDR5	ThermoFisher-eBioscience
hFoxp3 (healthy samples)	236A/E7	ThermoFisher-eBioscience
hCD56 (healthy samples)	CMSSB	ThermoFisher-eBioscience
hCD14 (healthy samples)	61D3	ThermoFisher-eBioscience
hCD3 (healthy samples)	SK7	BioLegend
hCD25 (healthy samples)	M-A251	BioLegend
hCD19 (healthy samples)	HIB19	BioLegend
hCD4 (ovarian cancer and human cells in NSG samples)	RPA-T4	BD Biosciences
hCD25 (ovarian cancer and human cells in NSG samples)	M-A251	BD Biosciences
hCD127 (ovarian cancer and human cells in NSG samples)	HIL-7R-M21	BD Biosciences
hCD8 (ovarian cancer and human cells in NSG samples)	RPA-T8	BD Biosciences
hOX40 (ovarian cancer and human cells in NSG samples)	ACT35	BD Biosciences
isotypes		Corresponding companies
fixable eFluor780 Live/Dead stain		ThermoFisher-eBioscience
aqua live/dead viability stain		ThermoFisher-Invitrogen
